# Acculturation and market integration are associated with greater trust among Tanzanian Maasai pastoralists

**DOI:** 10.1017/ehs.2021.10

**Published:** 2021-02-10

**Authors:** Aaron D. Lightner, Edward H. Hagen

**Affiliations:** Department of Anthropology, Washington State University, Pullman, WA, USA

**Keywords:** prestige bias, risk, cultural transmission, trust, acculturation, market integration

## Abstract

Acting on socially learned information involves risk, especially when the consequences imply certain costs with uncertain benefits. Current evolutionary theories argue that decision-makers evaluate and respond to this information based on context cues, such as prestige (the *prestige bias model*) and/or incentives (the *risk and incentives model*). We tested the roles of each in explaining trust using a preregistered vignette-based study involving advice about livestock among Maasai pastoralists. In exploratory analyses, we also investigated how the relevance of each might be influenced by recent cultural and economic changes, such as market integration and shifting cultural values. Our confirmatory analysis failed to support the prestige bias model, and partially supported the risk and incentives model. Exploratory analyses suggested that regional acculturation varied strongly between northern vs. southern areas, divided by a small mountain. Consistent with the idea that trust varies with socially transmitted values and regional differences in market integration, people living near densely populated towns in the southern region were more likely to trust socially learned information about livestock. Higher trust among market-integrated participants might reflect a coordination solution in a region where traditional pastoralism is beset with novel conflicts of interest.

## Graphical abstract

**Figure:**
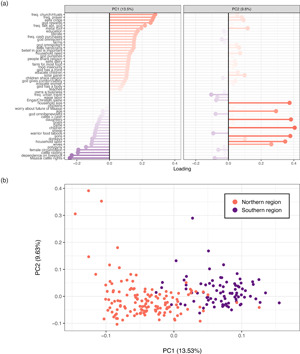

**Social media summary:** Trusting advice from others can involve risks of misinformation. This study supports the idea that people are wary of this risk in advice-giving scenarios, and that market expansion is associated with higher trust.

## Introduction

Individuals must often make critical decisions based on information provided by others who might be untrustworthy, either because their information is poor or they have incentives to deceive. As an example, suppose that a herder suggests to another where he should move his livestock during the dry season to find grass and water. In a semi-arid ecology such as northern Tanzania, this advice implies an unavoidable cost (moving the herd to another area) with a large but uncertain benefit. How should the herder decide if this advice is trustworthy? (Here, we define ‘trust’ as ‘reliance upon [socially learned] information … about uncertain environmental states and their accompanying outcomes in a risky situation’; Schlenker, Helm, & Tedeschi, 1973, p. 419; see also Yamagishi, Kikuchi, & Kosugi, 1999.)

Current theories of social learning focus on the source of information and/or risks of acting on the information. Some theories emphasise evolved learning biases, triggered by cues such as the prestige of the information source (Henrich, 2017; Richerson & Boyd, 2005), which we refer to as the *prestige bias model* (PBM). Other theories emphasise flexible copying based on incentives, i.e. expected outcomes of acting on the information and possible conflicts of interest with the information source (Binmore, 2011; Mercier, 2020; Morin, 2015), which we refer to as the *risk and incentives model* (RIM). In a preregistered study, we test the PBM and the RIM among Maasai pastoralists. Evaluating socially learned information is further complicated when individuals traverse varying cultural and economic contexts: individuals who might be trusted sources of information in one context might be mistrusted in another. We investigate these effects in a *post hoc* exploratory analysis.

### Prestige bias model of trust

In the simplest models of social learning, individuals simply learn from a random individual in the population (Rogers, 1988). Social learning can be enhanced, however, by preferentially copying more knowledgeable individuals. One strategy would be to assess the knowledge of all group members via personal experience over time, and then choose to copy the most knowledgeable individual(s). However, this would be time consuming and error prone – *directly* observing performances can be noisy, leading a learner to misperceive competence (e.g. see Boyd & Richerson, 1985, pp. 92–94, ch. 8). Alternatively, dual inheritance theorists argue that evolved context biases can solve this problem by exploiting simple and *indirect* social cues, triggering simple decision rules (Richerson & Boyd, 2005). Prestige bias involves preferentially copying individuals with *prestige* gained by ‘freely conferred deference’ (Boyd & Richerson, 1985; Henrich & Gil-White, 2001). This is efficient because it simplifies a complex learning task into a much simpler one. Relying on such a cue can reduce noise by ‘averag[ing] over many performances, which can help reduce the error in the learner's assessment of who to learn from’ (Henrich & McElreath, 2007, p. 559; see also Hill & Kintigh, 2009). Prestige bias is also adaptive because this simplification can be trusted across socioecological contexts and generational time (Henrich & McElreath, 2003). Modelling studies demonstrate that prestige can signal locally relevant skills and/or expertise (Plourde, 2008), and naive learners can trust prestige signals to acquire locally adaptive knowledge (‘information goods’) quickly and accurately in a wide range of conditions (Panchanathan, 2010). As Henrich et al. (2001) explain (p. 345, emphasis added):

A substantial amount of cross-cultural ethnography (e.g. Dove, 1993; Hammel, 1964; Rogers, 1995; Moore, 1957) and laboratory psychology (for a summary, see Gil-White and Henrich, 1999) suggests that humans everywhere possess a tendency to copy prestigious individuals, i.e. those who receive the most displays of respect/deference from others. This mechanism embodies two shortcut heuristics. First, by preferentially copying a ‘bundle’ of cultural traits from prestigious individuals (prestige correlates with skill/knowledge and often wealth) copiers can rapidly acquire a repertoire of fitness-enhancing or success-oriented traits (i.e. better-than-average solutions to the problems of life). Second, *rather than gradually learning via individual experience who the most successful, knowledgeable, or skillful individuals are, copiers rely on honest ethological and sociolinguistic signals of respect that other individuals display toward such high status individuals*.

Empirical support for the PBM is mixed (Jiménez & Mesoudi, 2019). In support, food taboos among pregnant and breastfeeding women in Fiji largely improved their health outcomes, and some of these taboos were transmitted by prestigious elderly women (Henrich & Henrich, 2010; cf. Placek, Madhivanan, & Hagen, 2017). Prestige was also a reliable indicator of hunting skill among the Hadza (Stibbard-Hawkes, Attenborough, & Marlowe, 2018) and Tsimane (von Rueden, Gurven, & Kaplan, 2008), although for the latter, ethnobotanical knowledge did not predict prestige (Reyes-Garcia et al., 2008). In experiments, children and adults use prestige cues to improve their performance in a novel task, especially when they are performing poorly (Atkisson, O'Brien, & Mesoudi, 2012; Chudek, Heller, Birch, & Henrich, 2012). Experiments have also found that when cues of success are available, participants will favour those cues over prestige cues (Brand, Heap, Morgan, & Mesoudi, 2020). Surveys of the ethnographic literature on social learning among hunter–gatherers and on leadership, however, found little evidence of prestige-biased learning (Garfield, Garfield, & Hewlett, 2016; Garfield, Hubbard, & Hagen, 2019).

Because the PBM relies on a narrow, restricted range of cues, a cost–accuracy tradeoff leaves room for costly or ‘irrational’ behaviours with specific, unavoidable, maladaptive side effects (e.g. see Richerson & Boyd, 2005, pp. 119–124, 156 for discussion). In weaker versions of the PBM prestige is conceptualised as one important cue among many, whereas in stronger versions of the PBM prestige can override other cues and decisions thus sharply diverge from individual self-interests, including non-adaptive food taboos (Aunger, 1994; Henrich & Henrich, 2010), market bubbles (Bell, 2013) and suicide epidemics (Henrich & McElreath, 2007; Mesoudi, 2009). This ambiguity among possible interpretations in the prestige-bias literature is discussed in Morin (2016).

### Risk and incentives model of trust

People might also be ‘epistemically vigilant’, or largely resistant to social influence while conditionally trusting advice based on message content, risk, incentives and perceived conflicts of interests with the sender (Mercier & Sperber, 2017; Trouche, Johansson, Hall, & Mercier, 2018; see also Binmore, 2011; Hess & Hagen, 2006; Mercier, 2020; Morin, 2015). If the trustworthiness of socially learned information is questionable, the RIM emphasises that acting on it is a *gamble* between two options, possibly with equivalent expected values, with a low-variance safe option (high probability of receiving a low payoff) and a high-variance risky option (lower probability of a high payoff). Individuals preferring the safe option are *risk averse*, and those preferring the risky option are *risk seeking*.

Which of these option types is adaptive depends strongly on an organism's current state: Foragers with a sufficient energy budget, for example, should be risk averse, whereas foragers with a dangerously low energy budget should be risk seeking (Stephens, 1981). The relationship between resource scarcity and risk seeking, mediated by stress, is supported in non-human animal experiments manipulating energy budgets (Caraco et al., 1990; Kacelnik & Bateson, 1996), as well as observational studies in humans (see Winterhalder, 2007 for review). As biologists and economists have observed, this apparent risk sensitivity of decision-making might be explained as maximising long-term growth rates under multiplicative dynamics (Kacelnik & Bateson, 1996; Peters, 2019; Peters & Gell-Mann, 2016; Price & Jones, 2020).

Theoretical distinctions between social vs. individual learning strategies could distract from the fundamental task in most real-world decision-making: weighing the expected costs and benefits (Morin, 2015). If acting on social influence is cheap and outcomes are trivial, then a useful decision rule should not seek more expensive cues, but if the stakes are high enough, then a high cost for accuracy might be worth paying.

Experimental evidence has supported some key aspects of the RIM in humans. People are more likely to take high-risk decisions under stress and resource scarcity (Dalton, Nhung, & Rüschenpöhler, 2019; Kirchler et al., 2017; Putman, Antypa, Crysovergi, & van der Does, 2009), although some experiments show that poverty induces risk aversion (a poverty trap; Yesuf & Bluffstone, 2009). Kuznar (2001) also showed that higher levels of wealth were associated with risk aversion among moderately wealthy herders, but with the exception of risk-prone herders in the highest wealth class. In social contexts, participants’ evaluations of argument persuasiveness are conditioned on how relevant the consequences of its message would be for them (Petty & Wegener, 1998). If consequences are not relevant, then people rely on social information and heuristics such as expertise and audience approval (Axsom, Yates, & Chaiken, 1987). If they are relevant, then they evaluate the content of the message (Petty, Cacioppo, & Goldman, 1981). Content evaluation might trend towards psychologically attractive ideas (Miton, Claidière, & Mercier, 2015), individual preferences (Acerbi & Tehrani, 2018) or attempts to reduce the ambiguity of social cues when multiple cues are available (Conway & Schaller, 2005). People are sensitive to conflicts of interest and social informational ‘dependencies’ (Hess & Hagen, 2006; Mercier & Miton, 2019), and are more likely to trust expert advice when they are given clear demonstrations of expertise rather than an argument from expertise (Mercier et al., 2019).

### The impact of changing ideational and material culture on trust

Another perspective, which is consistent with the RIM and some interpretations of the PBM, is that decisions about social information can flexibly adapt to variation in ‘ideational’ (values and norms) and ‘material’ (economic) culture. If widespread incentives are suddenly distorted by changing material conditions, such as market integration and/or developing infrastructure, then ideational changes might predictably follow (Aoki, 2011; Binmore, 2011; Yamagishi & Suzuki, 2009). Proponents of this view often start from an assumption of *methodological individualism*, similar to the RIM (i.e. social phenomena are grounded in individual incentives; see North, 1990). Market integration in developing nations and small-scale societies imposes novel transaction costs, which can in turn disrupt existing sharing institutions and undermine widespread trust (e.g. Ensminger, 1992; Baird, 2014; Kasper & Borgerhoff Mulder, 2015). This might render social status, kinship and reciprocity insufficient for establishing trust in most social interactions. This would create a demand for culturally evolved norms to sustain mutually beneficial exchanges, such as fairness and/or religious beliefs that stabilise trust by manipulating perceived incentives (Henrich et al., 2010) or encourage the use of inferred mental states in moral judgements (Curtin et al., 2020). Costly religious rituals also signal trustworthiness among strangers (Ensminger, 1997; Power, 2017), and religious beliefs in omniscient, moralistic gods stabilise trust in large-scale, market-integrated communities (Lang et al., 2019; Purzycki et al., 2016).

## Study aims and context

Here, we (a) test the PBM and RIM as models of trust using a vignette-based experiment involving advice about livestock among Maasai pastoralists and (b) conduct an observational study of the impact of recent cultural and economic changes, such as market integration and shifting cultural values, on trust.

### Preregistered predictions

We preregistered predictions for strong and weak versions of the PBM, and for the RIM. Our prediction for both the strong and weak versions of the PBM model was that: (a) advice about livestock would be more likely to be trusted and acted on when it comes from a prestigious person than when it comes from a person deemed generally knowledgeable from personal experience. Our prediction for the strong version only was that (b) trust would not be impacted by material incentives, such as household resource scarcity or livelihood diversification (i.e. how much they depend on livestock for subsistence).

Our predictions for the RIM were that: (a) advice would be more likely to be trusted when resources are scarce (i.e. participants are more likely to take a risk) and less likely to be trusted when a participant is wealthy and mostly depends on livestock for subsistence (i.e. participants are more risk averse). Additionally, it predicts (b) no additional effect of prestige cues on trust over other social cues, such as knowing from experience that someone is generally knowledgeable.

Our prediction for the weak version of the PBM only was that advice would be more likely to be trusted when it comes from a prestigious person and when resources are scarce (PBM+RIM).

Preregistration materials can be viewed at https://osf.io/5p7ut.

### Description of the field site

This study took place in Eluwai, a Kisongo Maasai village in Monduli Juu highlands of northern Tanzania. (In Tanzania, ‘villages’ refer to administrative jurisdictions, and do not necessarily imply that households in the community are clustered together.) Kisongo Maasai groups in Monduli Juu have depended mainly on cattle for centuries. Rainfall occurs bimodally and consists of short, massive downpours separated by long, hot dry seasons. Maasai have traditionally been semi-nomadic, patterning seasonal movement with expected rainfall while navigating livestock risks, such as drought and disease (Jacobs, 1965; Spear & Waller, 1993). Strategies for reducing risk can include manipulating herd composition and breeding rate in ways that maximise long-term household survival (Dahl & Hjort, 1976; Mace, 1993), and avoiding energetically expensive migrations into overgrazed or excessively dry areas (Butt, 2016). Cattle herding is a high-risk livelihood, and in a semi-arid ecology such as Monduli Juu, a successful herder is a risk averse and mobile herder.

In the present day, however, people in Monduli Juu are almost completely settled into sedentary lifestyles, a result of postcolonial land privatisation and the Ujamaa villagisation initiative that divided rural regions into administrative jurisdictions termed ‘villages’ (Boesen, 1976). Land conflict and overgrazing now make pastoralism an exceedingly difficult subsistence strategy (McPeak, Doss, & Little, 2011). The last two decades or so have seen a sharp uptick in agricultural practices, land privatisation, spreading urbanisation and infrastructure development. Now, more than ever before, herd movements are restricted by property lines, and the grass and water on which livestock rely are scarce resources. These changes are accompanied by market integration and a steady influx of cash from safari tourism, non-government organisations investing in formal education, and increasingly influential local Christian missionaries (Hodgson, 2005). As a result, there is some tension between traditional vs. modern lifeways: Maasai value their traditions and pure reliance on cattle is considered an ideal, but a growing number of Kisongo Maasai see ongoing cultural and economic changes as opportunities they should embrace (Heckelsmiller, 2015; Hodgson, 1999; see also Galaty, 1982; Homewood, Trench, & Kristjanson, 2009; Jandreau & Berkes, 2016).

**Figure 1.**
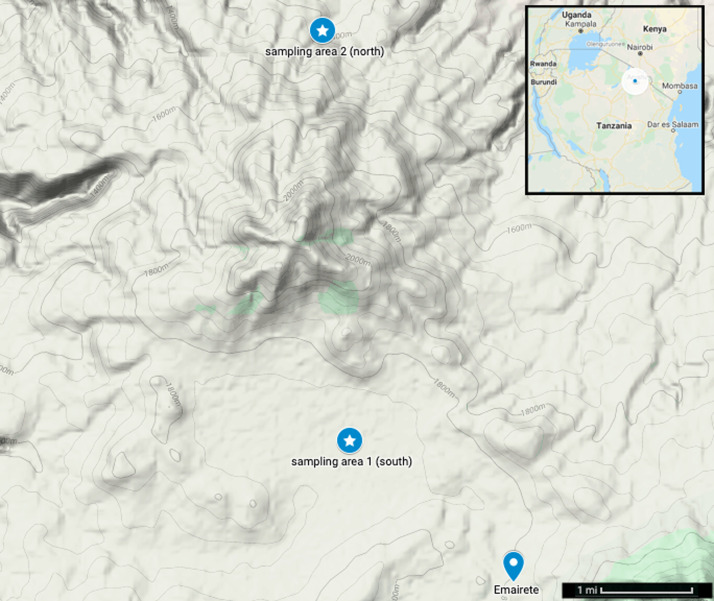
Eluwai village area with terrain image showing the approximate center of sampling area 1 (southern region) and sampling area 2 (northern region), both of which are separated by a small mountain (center). Emairete town neighbors the south of sampling area 1, and is connected by paved road to a larger town, Monduli Chini, which is slightly further south (not included in this map). Inset: Map of Tanzania showing the approximate location of the fieldsite in northern Tanzania (blue point, encircled in white).

Eluwai village spans a wide range of rural landscapes in Monduli Juu, and is roughly split into northern and southern regions by a forested mountain, about 600 metres in height (average base to peak; see Figure 1). The southern region is connected by a walking path to Emairete, a small but densely populated town with a weekly market, multiple churches and a few small businesses. Cell phone communication in the southern region is both possible and frequent, and Emairete has an Airtel retailer for purchasing cell phone minutes. Emairete itself is linked by paved road to Monduli Chini, a much larger town nearby consisting of several businesses and biweekly markets. The northern region, in contrast, is relatively isolated, surrounded by sparsely populated highlands and the Rift Valley running along the northeast. Cell phone reception is mostly lacking. Contact from the northern to southern region can require about a day or so of walking during the dry season, but is difficult when walking routes and erosion canals are flooded in the rainy season.

## Methods

Data collection involved structured surveys and a trust vignette experiment with adult Kisongo Maasai pastoralists (*N* = 225; 41% female, 59% male) in both northern (*N* = 141) and southern (*N* = 84) regions of Eluwai. Surveys in the southern region were collected by A.D.L. with assistance from a Maasai translator, and by an additional local Maasai research assistant. Surveys in the northern region were collected by another local Maasai research assistant. Both research assistants have more than 10 years of experience administering surveys to local populations, and were trained to conduct the survey by A.D.L. Data were collected from January to March 2020. Interviews took about 30 minutes. Each participant was paid 10,000 Tanzanian Shillings (about US$4.35) for their participation (about the price of lunch at a local restaurant). All preregistered predictions, models and analysis scripts can be found at https://osf.io/5p7ut. All protocols and survey materials were approved by Washington State University IRB and Tanzanian Commission for Science and Technology (COSTECH) prior to data collection.

### Study design

To test the PBM and RIM, we conducted a vignette experiment in which a hypothetical person from the community describes an inconveniently faraway location (about a day walking), where he says the participant should move their livestock to find plenty of available grass and water. The advice presents a conundrum: should the participant trust the advice and act on it? Should they be sceptical and fact-check it first? Should they reject the advice altogether? If the advice is accepted, then it will lead to a large benefit if true, but a large cost if false. If it is rejected, then it will be an opportunity cost if true, but avoid a large cost if false. If the advice is fact-checked before acting on it, then a smaller cost is taken on to reduce the risk of accepting the advice and acting on it. (In the literature on the evolution of social learning, asocial learning is *a priori* more costly than social learning. It is worth emphasising that our study does not compare social with asocial learning. Instead, it compares social to state-dependent learning, with asocial learning as one of our two outcomes (i.e. the ‘fact-checking’ outcome variable), consistent with the literature we cite on trust. In other words, *given social learning*, what predicts trust – prestige or state?)

Each participant was randomly assigned to either a *prestige* condition or a *participant experience* condition. In the prestige condition (*N* = 113) the source of advice was described as a person with high levels of *nkanyit*, an important Maasai prestige concept that translates in Maa to ‘respect’, but also has connotations of fear and deference, cattle wealth and indisputable authority (Spencer, 1965, 2004b). To confirm these connotations, we asked a subset of our participants to freelist what gives a person nkanyit. The most salient responses included cattle wealth, caring for a large family, having good moral character, helping others and being knowledgeable (see the Supporting Information). Consistent with the assumptions in our study design, informants also emphasised that, although nkanyit can imply knowledge, knowledge does not imply nkanyit.

Prestige bias theorists argue that cues of prestige can be more reliable than ‘gradually learning via individual experience who the most successful, knowledgeable, or skillful individuals are’ (Henrich et al., 2001, p. 345). In the participant experience condition (*N* = 107) the source of advice was therefore described as someone the participant has known from personal experience to be generally knowledgeable. (Our use of the term *experience* refers to the participant's experience that the fictional advisor is generally knowledgeable, and does not imply that the fictional advisor actually has experience of the grazing conditions that he is describing.)

Participants were then asked how much they trusted the advice, and whether or not they would fact-check it first (i.e. personally visit before taking their livestock there). A more comprehensive structured survey was then conducted (described below). It is worth emphasising that in neither condition was the fictional source of advice described as having specific or direct knowledge of grazing conditions. See the Supporting Information for complete vignette text and nkanyit freelist data.

### Measures

#### Experimental outcomes

Our two post-intervention outcome variables were *trust* (stated level of belief that the advice given is true) and *fact-checking* (if the participant would verify the advice before acting on it). Trust outcomes were coded on a three-point scale (1 = completely trust, 0.5 = somewhat trust, 0 = does not trust). Fact-checking outcomes were measured as simple yes/no responses (1 = yes, 0 = no). See the preregistration https://osf.io/5p7ut and Section 3 of the Supporting Information for details.

#### Observational measures for preregistered tests

Household-level resource scarcity was based on food insecurity scores and a proxy measure of household need. Food insecurity scores were determined by a modified five-item version of a standard six-item household food insecurity survey, where higher values indicate higher insecurity (Blumberg, Bialostosky, Hamilton, & Briefel, 1999). (Prior to data collection, a question about diet breadth was removed because it did not make sense for participants in this region, where narrow diets of milk and meat are ideal.) Household need was approximated using consumer-to-producer ratios (i.e. total number of people living in the household, divided by people reported to regularly contribute to subsistence in the household; more consumers per producer implied higher need). Measures of household wealth were based on an index consisting of three reliable wealth indicators in the region: presence/absence of a solar panel (1 = presence, 0 = absence), roof material (1 = metal, 0 = grass) and number of wives in the household. To measure how dependent a household was on livestock, we collected a list of the different ways in which people in the household made a living, using freelists and prompted options with yes/no responses. Prompts were livestock, farming, milk/meat sales, crop sales, handcraft sales, wage labour, owning a business, teaching and other (if yes, specify). Dependence on livestock was then estimated by dividing presence/absence of herding livestock for subsistence (1 = yes, 0 = no) by the total number of subsistence sources listed, creating a proportion of livelihood strategies involving livestock (1 = completely dependent on livestock, 0 = not dependent on livestock at all).

#### Exploratory measures

Our survey included several measures across two domains – ideational and material – for which we had no preregistered hypotheses. Measures of traditional beliefs (TB) included cultural values, such as religious beliefs and practices, e.g. religious affiliation, frequency of prayer (coded on a ranked scale between 1 = never and 5 = very often) and beliefs about god's characteristics. Whether or not god punishes misbehaviour; rewards good behaviour and is omniscient, omnibenevolent and/or omnipotent were each measured as yes (1), no (−1), or don't know (0). Cultural values involved agree/disagree responses to divisive statements that are rooted in traditional Maasai ideals. Traditionally agreeable statements include: females should be circumcised, all cattle in the world rightfully belong to Maasai people, it is acceptable to raid cattle from people who are not Maasai and it is ideal for elder men to have multiple wives. A disagreeable statement includes: it is acceptable for women to see a warrior eat meat. Traditionally neutral statements held mostly by Christians in the region include: belief in god is the most important thing in life, and women and children should be educated in school (e.g. Jacobs, 1965; Hodgson, 1999; Spear & Waller, 1993; Spencer, 1965). Responses to each statement in the cultural values survey were measured as strongly agree (2), agree (1), no opinion (0), disagree (−1), strongly disagree (−2).

Material domains included an *a priori* index of market integration (MI) to approximate frequency of cash sales and purchases, based on how often people made purchases at the market (coded on a ranked scale between 1 = never and 5 = very often), whether or not participants sold handcrafts, crops and/or dairy products at markets (0 = no, 1 = yes for each) and frequency of cell phone use (1 = never, 2 = sometimes, 3 = often), yielding an index range of 2–10. Measures also included level of education (0 = none, 1 = primary, 2 = secondary) and literacy (0 = no, 1 = yes). Herd size and composition (e.g. cattle, sheep, goats, donkeys and chickens) were self-reported and also included as tropical livestock units, an estimate of livestock resources based on grazing capacity (Jahnke & Jahnke, 1982).

Although our use of nkanyit as a prestige cue was motivated by prior key informant interviews and existing literature (e.g. Spencer, 1965, 2004a, b), we also collected freelist data (*N* = 57; south, *N* = 41, north, *N* = 16) about nkanyit to validate this choice. See Supporting Information for details.

### Confirmatory analyses

We tested our predictions using separate sets of logistic regression models for the PBM and the RIM, as specified in our preregistration, with *α* = 0.05. For the strong version of the PBM, our independent variable was the vignette condition only (*VC*: 0 = experience, 1 = prestige). To adhere to our preregistration, we modelled both outcomes using logistic regression, despite the trust outcome being on a three-point scale (0, 0.5 and 1; see Britt & Weisburd (2010) and the Supporting Information where we fit ordinal regression models). We predicted a statistically significant positive coefficient for *VC* for the trust outcome, and a statistically significant negative coefficient for the fact-checking outcome:
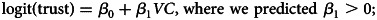

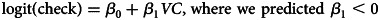
For the RIM, our independent variables were food insecurity scores (*F*), household need (*N*), wealth (*W*) and dependence on livestock (*D*) for subsistence. We predicted that for trust outcomes aggregated across conditions (i.e. ignoring any effect of *VC*), we would find statistically significant positive coefficients for *F* and *N*, and statistically significant negative coefficients for *W* and *D*. We predicted these coefficients to be reversed for fact-checking outcomes:
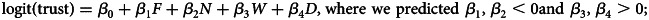

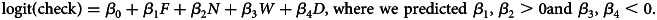
We then compared the PBM, RIM and PBM + RIM (PBM + RIM comprised the RIM models with an additional term for *VC*, which corresponds to the weak version of the PBM) using the corrected Akaike information criterion (AICc), preferring the model with the lowest AICc value (Burnham & Anderson, 2004).

### Exploratory analyses

Prior to fieldwork, we anticipated that cultural and economic variation would be associated with different response patterns but did not know how it would be distributed. To explore covariation of all diverse variables characterising sociodemographic, economic and ideational aspects of participants in our dataset, we conducted a principal components analysis (PCA) on all quantitative observational measures on households and participants for which there were 10 or fewer missing values, resulting in 53 measures across all domains in the survey. If the principal components were interpretable, we aimed to test if one or more of them was associated with our *trust* and *fact-checking* outcomes. (The PCA excluded both outcome variables, region, and experimental condition.)

To use data from all participants, we imputed missing values using the *mice* package (van Buuren & Groothuis-Oudshoorn, 2011) for multiple imputation by chained equations (Azur et al., 2011), with the default predictive mean matching method for numeric and logistic regression for binary variables. MICE assumes that data are missing at random. That is, after controlling for all other variables in the study, any remaining missingness is completely random. All exploratory results, including the PCA, are pooled estimates from five imputed datasets (Rubin, 1988). See the Supporting Information for a walkthrough of variable selection, multiple imputation processes and quality checks on imputed datasets. (Because we did not preregister imputation, we did not use it for the confirmatory analyses.) Two participants had extremely high numbers of children, which had an undue influence on the PCA, and were therefore removed from the exploratory analyses.

## Results

### Cultural and regional variation

**Figure 2.**
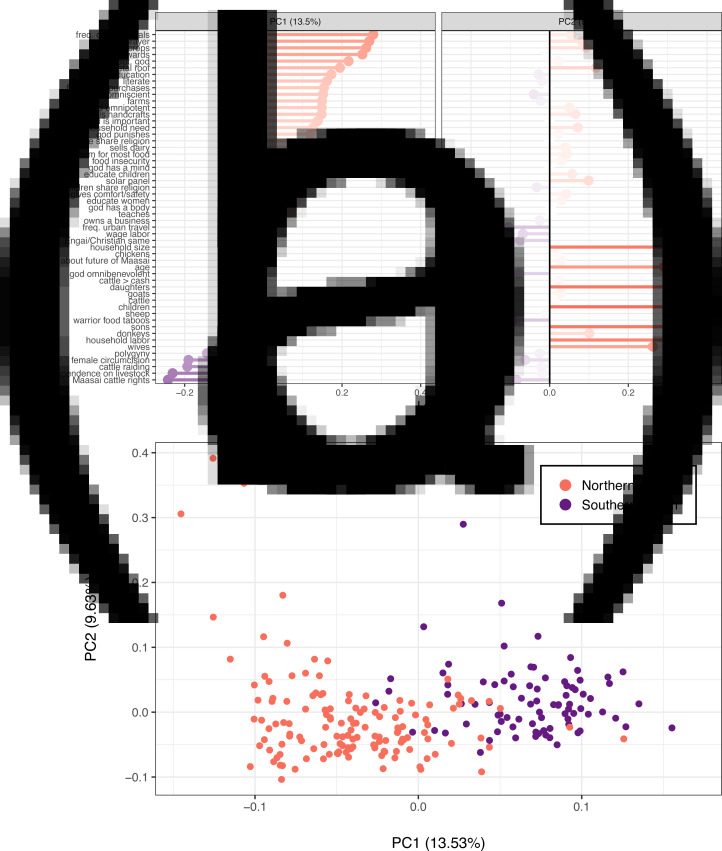
A: PCA loadings on PC1 and PC2, after including 53 quantitative variables from diverse domains in our analysis. PC1 corresponds to a latent variable characterizing acculturation vs. traditional practices and beliefs. PC2 corresponds to a latent variable characterizing household size. B: PCA biplot, with each point representing one participant. Point colors correspond to participant region.

Summary statistics are provided in Table 1. PCA results showed systematically different response patterns corresponding to ideational, material and regional variation around Eluwai. The variables with high negative loadings on PC1 exclusively represented adherence to traditional Maasai ideals, beliefs and material practices (large herds; high dependence on livestock; approval of cattle raiding, female circumcision and polygyny; and agreement with traditional Maasai beliefs about cattle ownership). The variables with high positive loadings on PC1 represented adherence to more recently introduced ideals, beliefs and material behaviours, such as crop sales, farming, higher education, literacy, handcraft sales and prayer frequency (prayer frequency is generally higher among Christians, mean = 3.6, than among traditional Maasai believers, mean = 2.5; *t =* 5.1, *p =* 10^−6^). PC2 reflected household size (see Figure 2a).

**Table 1.** Summary statistics for most of the quantitative and ranked observations data used in this study. This includes data used to model and test our study predictions, but also includes descriptive variables about the sample and a few key variables systematically varying across different regions of the field site. Trust and check refer to our two outcome variables, and food insecurity, household need, wealth and dependence on cattle were used as observed predictors. Excluding both outcome variables, each variable showed here was included in the principal components analysis described in the text

Name	Complete	Mean	Standard deviation	Range	Histogram	Name	Complete	Mean	Standard deviation	Range	Histogram
Age	0.99	42.3	16.0	19–80	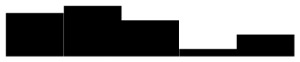	Polygyny	1.00	0.8	1.1	−2–2	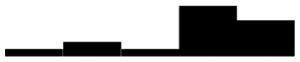
Wives	0.98	2.3	2.0	0–12	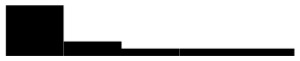	Warrior food taboos	0.98	−0.5	1.2	−2–2	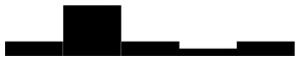
Children	0.98	6.5	6.8	0–40	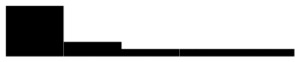	Cattle raiding	0.96	−0.1	1.3	−2–2	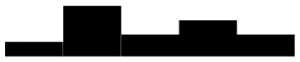
Literate	0.99	0.2	0.4	0–1	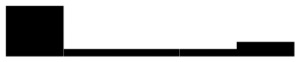	Educate children	1.00	1.2	0.9	−2–2	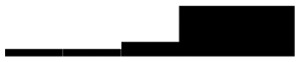
Education	0.99	1.3	0.5	1–3	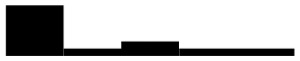	Educate women	0.97	0.9	1.0	−2–2	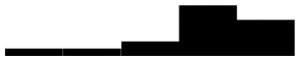
Sells dairy	0.98	0.1	0.3	0–1	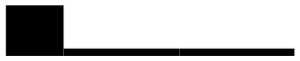	Cattle > cash	0.99	0.7	1.1	−2–2	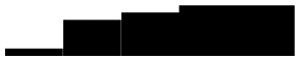
Sells handcrafts	0.98	0.1	0.3	0–1	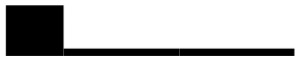	Belief in god is important	0.98	1.0	0.9	−2–2	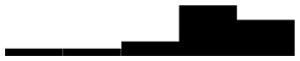
Wage labour	0.98	0.1	0.3	0–1	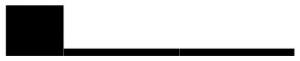	Children share religion	0.98	0.9	1.0	−2–2	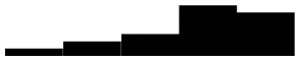
Farms	0.98	0.8	0.4	0–1	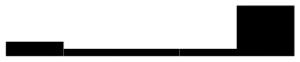	People share religion	0.98	0.8	1.0	−2–2	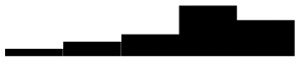
Sells crops	0.98	0.4	0.5	0–1	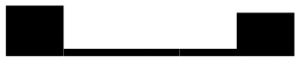	Farm for most food	0.99	1.0	0.9	−2–2	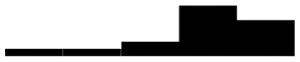
Owns a business	0.98	0.2	0.4	0–1	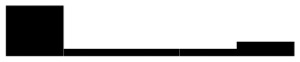	Female circumcision	0.98	0.3	1.3	−2–2	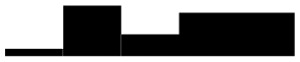
Teaches	0.98	0.0	0.2	0–1	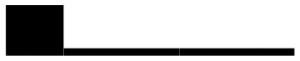	Worry about future of Maasai	0.96	0.3	1.1	−2–2	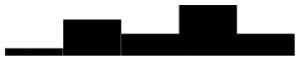
Household size	0.98	9.2	10.8	1–105	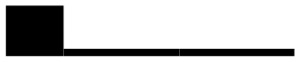	God gives comfort/safety	0.99	1.1	0.9	−2–2	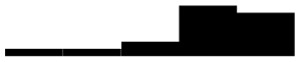
Household labour	0.98	4.5	8.7	1–100	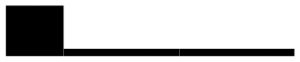	Donkeys	0.98	6.0	11.7	0–92	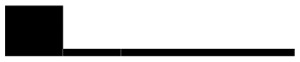
Frequent urban travel	0.99	1.8	1.1	1–5	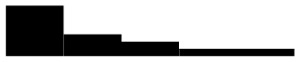	Chickens	0.99	5.3	19.0	0–250	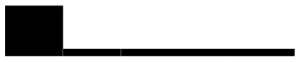
Engai/Christian same	1.00	0.6	0.7	−1–1	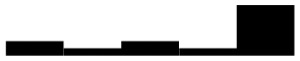	Cattle	0.98	34.7	90.6	0–1000	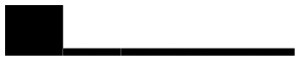
God has a mind	0.99	0.2	0.8	−1–1	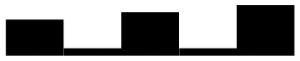	Goats	0.97	32.5	67.3	1–750	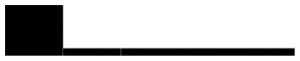
God has a body	0.99	−0.2	0.8	−1–1	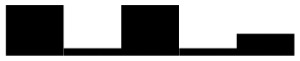	Sheep	0.97	26.4	59.6	0–520	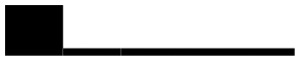
God omnipotent	0.99	0.8	0.5	−1–1	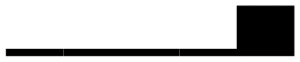	Metal roof	1.00	0.2	0.4	0–1	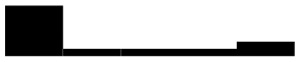
God omniscient	1.00	0.8	0.5	−1–1	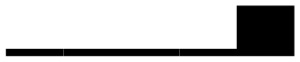	Solar panel	0.97	0.3	0.4	0–1	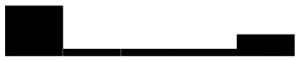
God omnibenevolent	0.99	0.8	0.6	−1–1	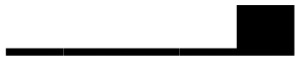	Market integration	0.96	4.1	1.2	1–7	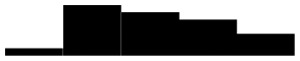
God punishes	0.99	0.3	0.7	−1–1	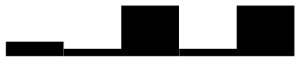	Food insecurity	1.00	1.0	0.4	0–1.75	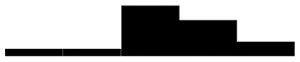
God rewards	0.98	0.5	0.6	−1–1	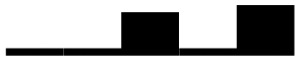	Household need	0.97	2.6	1.8	1–20	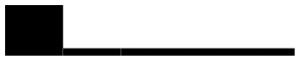
Frequent church/rituals	0.97	2.1	1.4	1–5	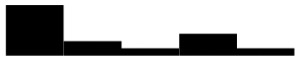	Dependence on livestock	0.97	0.5	0.2	0–1	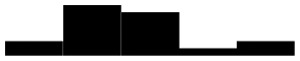
Frequent prayer	0.96	3.1	1.6	1–5	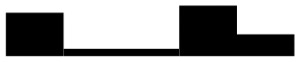	Frequent cash purchases	0.99	3.5	0.7	1–5	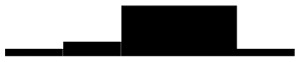
Frequent talk about god	0.96	1.5	1.1	1–5	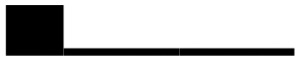	Trust	0.97	0.3	0.4	0–1	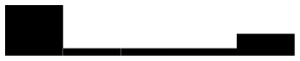
Maasai cattle rights	1.00	0.8	1.3	−2–2	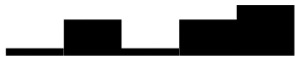	Fact-check	0.92	0.8	0.4	0–1	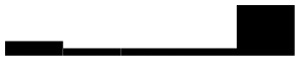

We therefore interpret PC1 as a latent *acculturation* variable corresponding to *both* ideational and material changes in the area (e.g. market integration, missionisation, education). (Acculturation is defined as an exchange of cultural features resulting from different cultural groups coming into continuous first-hand contact, such that cultural patterns of either or both groups might be changed and the groups remain distinct; see Kottak (2013, p. 569). Here, it refers to Tanzanian Maasai adopting ideas and behaviours that are a consequence of globalisation.) Ideational and material variation along PC1 largely mapped onto regional variation, such that participants living north of the mountain clustered along the lower end of PC1 (more traditional) and participants living south of the mountain (near town, markets, churches, paved roads, and schools) clustered along the higher end of PC1 (more acculturated) (see Figure 2b). We found no meaningful sex differences in our PCA results. See Supporting Information for details.

### Confirmatory analyses: testing the PBM and RIM

In both the vignette prestige condition and the experience condition, advice was treated with strong levels of scepticism (experience condition: 32% did not trust, 5.5% somewhat trusted and 11% completely trusted; prestige condition: 33% did not trust, 4.6% somewhat trusted and 14% completely trusted), and most participants stated that they would fact-check the advice before acting on it (86% in the experience condition, 82% in the prestige condition). Thus, participants had approximately equal, but low, trust for advice from both the prestigious individual and from the individual known to be knowledgeable from personal experience.

**Figure 3.**
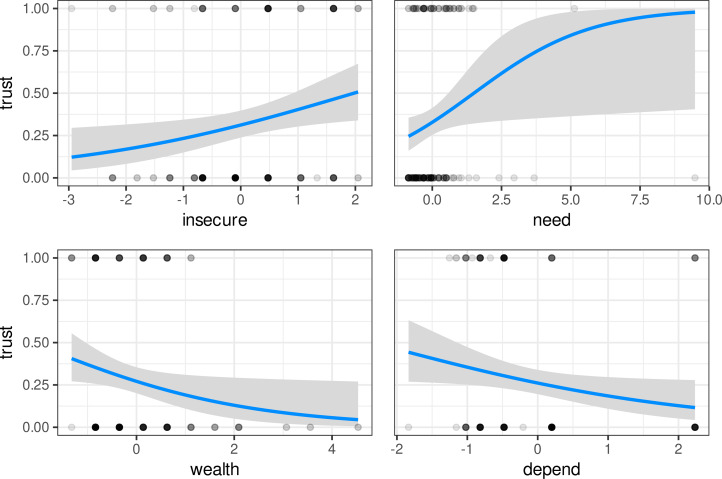
Logistic regression models for RIM predictors on trust outcomes. Model coefficients are in table 2 (column 2). Trust outcomes equal to 0.5 were rounded to 0 or 1 if their residuals were negative or positive, respectively.

In our confirmatory analyses for trust outcomes, the RIM was supported (see Table 2 for logistic regression model parameters and statistics, and Figure 3 for RIM effects plots). AICc model selection suggested that the RIM had better support than the PBM (strong version) and PBM + RIM (weak version). (We re-ran trust models using an ordered logistic regression and found similar effects in each of our models. See the Supporting Information for additional analyses and weighted AICc table.)

**Table 2.** Logistic regression models for trust outcomes (left three models) and fact-checking outcomes (right three models) based on condition, and on scaled measures of household food insecurity, need, wealth and dependence on livestock as a source of subsistence. Estimates are log odds, with standard error in parentheses. For each outcome variable, output is shown for preregistered models: prestige bias model (PBM), risk and incentives model (RIM) and PBM + RIM.

	*Dependent variable*					
	Trust			Check		
	PBM	RIM	PBM + RIM	PBM	RIM	PBM + RIM
Condition (prestige)	0.11		0.22	−0.32		−0.52
	(0.30)		(0.35)	(0.38)		(0.42)
	*p* = 0.70		*p* = 0.53	*p* = 0.41		*p* = 0.22
Insecure		0.40	0.40		−0.16	−0.15
		(0.17)	(0.17)		(0.20)	(0.20)
		*p* = 0.02*	*p* = 0.02*		*p* = 0.43	*p* = 0.45
Need		0.48	0.45		0.08	0.12
		(0.23)	(0.23)		(0.21)	(0.23)
		*p* = 0.04*	*p* = 0.05*		*p* = 0.70	*p* = 0.62
Wealth		−0.46	−0.45		0.30	0.28
		(0.22)	(0.22)		(0.25)	(0.25)
		*p* = 0.04*	*p* = 0.05*		*p* = 0.23	*p* = 0.26
Depend		−0.44	−0.47		0.36	0.44
		(0.21)	(0.22)		(0.26)	(0.27)
		*p* = 0.04*	*p* = 0.04*		*p* = 0.17	*p* = 0.11

*p < 0.05; **p < 0.01; ***p < 0.001.

### Exploratory analyses

#### Regional variation at the field site

**Figure 4.**
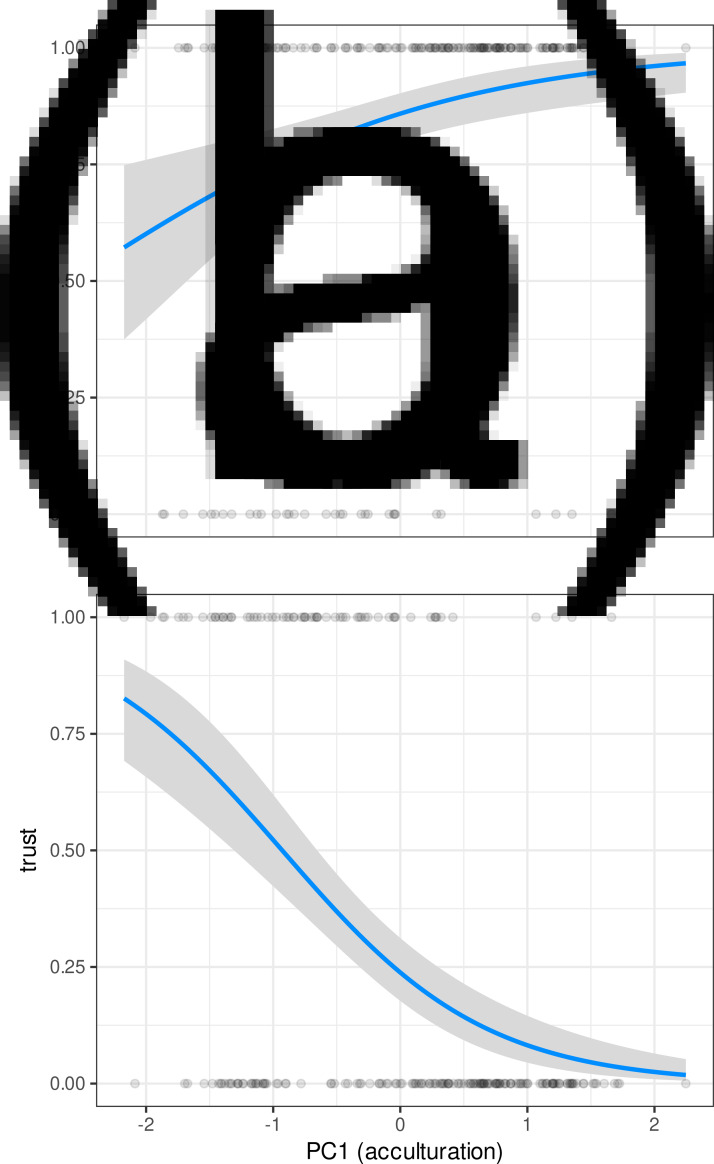
Fact-checking outcomes (A) and trust outcomes (B) predicted by PC1, the acculturation variable characterizing response patterns along the northern vs. southern sampling areas. Higher levels of PC1 correspond to higher levels of acculturation, such as Christianization and market integration. Lower levels of PC1 correspond to lower levels of acculturation, or traditional Maasai beliefs and economic practices. In (B), trust outcomes equal to 0.5 were rounded to 0 or 1 if their residuals were negative or positive, respectively.

We interpreted PC1 (Figure 2) to be a latent acculturation variable, which was systematically lower in the northern region and higher in the southern region. Responses in the northern vs. southern regions varied on trust outcomes (north: 1 = 51%, 0.5 = 24%, 0 = 25%; south: 1 = 7.8%, 0.5 = 1.4%, 0 = 86%) and fact-checking outcomes (north: 1 = 69%, 0 = 31%; south: 1 = 92%, 0 = 8%) (see Figure S7). We therefore modelled each outcome variable as a function of PC1. Consistent with regional patterns, more acculturated participants were more likely to trust livestock advice and less likely to fact-check it, whereas less acculturated participants were less likely to trust and more likely to fact-check (Figure 4).

#### Hierarchical cluster analysis

**Figure 5.**
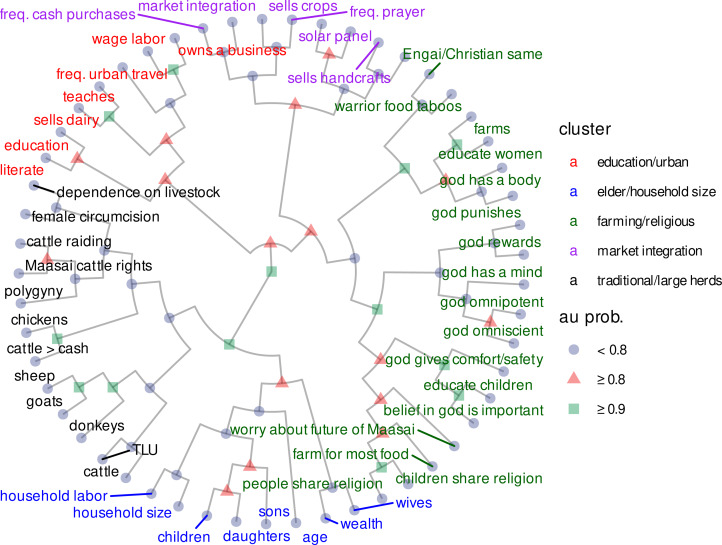
Hierarchical clustering dendogram with shapes corresponding to approximately unbiased (au) branching probabilities (bootstrapped n = 10,000), and colors corresponding to cluster ID. Each cluster is based in part on au probabilities and our interpretation of cohesive clusters (e.g., market integration, traditional livelihoods) Some clusters are less straightforward than others to interpret, but we nevertheless include a short cluster description next to each color.

Variables belonging to both ideational and material categories had high loadings on PC1 (Figure 2a), which in turn distinguished the northern and southern regions (Figure 2b). To explore if response patterns naturally formed ideational vs. material clusters, we conducted a hierarchical cluster analysis using the Ward agglomeration method, with distances as 1 − *corr*, and cluster *p*-values computed via multiscale bootstrap resampling (Suzuki, Terada, Shimodaira, & Suzuki, 2019). We identified five clusters that were reasonably well-supported by the bootstrap procedure (*p* > 0.8). These were education/urban, elder/household size, farming/religious, MI, and traditional beliefs/large herds (TB). See Figure 5. Two of these (TB and MI) were clearly interpretable as ideational vs. material. Because we also developed an *a priori* MI index (see ‘Exploratory measures’), we denote the MI cluster here as *empirically determined MI* (EMI). (Note that we made no *a priori* TB index, as we did with MI.)

**Figure 6.**
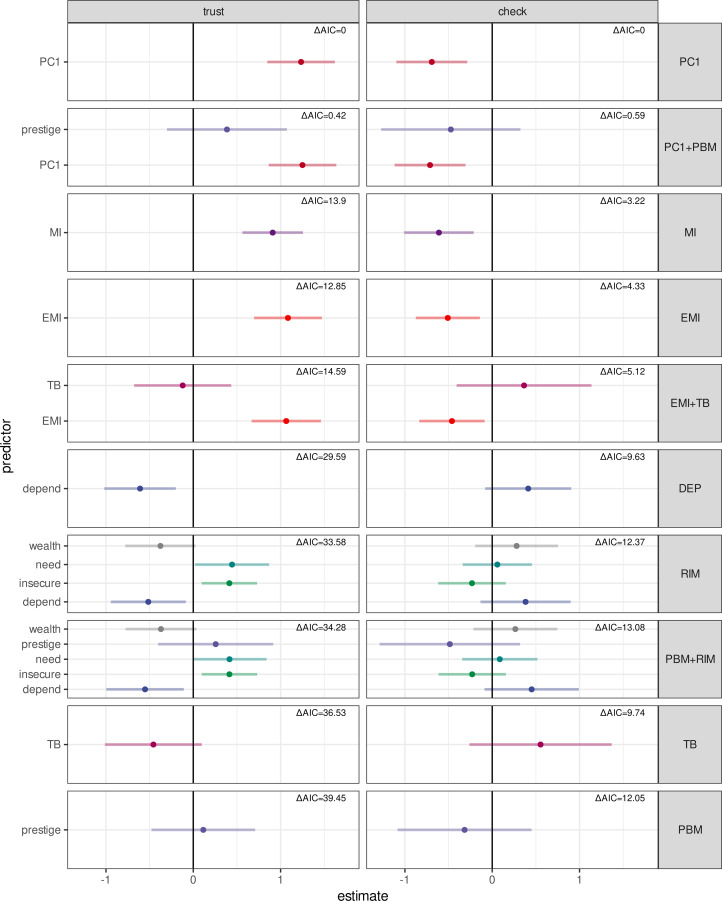
Coefficients plot for exploratory logistic regression models predicting trust and fact-checking outcomes. Points indicate regression coefficients (log odds scale), and error bars are +/- 2 SE. Colors correspond to different predictors included in various models, whereas facets separate models included in the model comparison in this section. Facets are ordered from top to bottom in order of AICc score in weighted model selection. Tables for this are included in the SI.

To explore if material or ideational clusters better predicted trust than PC1, we used MI, EMI, TB and dependence on livestock for subsistence each as separate predictors of trust and fact-checking outcomes. (We included a model with dependence on livestock (referred to in the RIM as *depend*) because it strongly correlated with PC1, and varied markedly by region. In Figure 6, this model is abbreviated as *DEP*.) Comparing these models with each other and the confirmatory models, we found that MI and EMI each predicted higher trust and lower fact-checking, and while these effects were larger than those in the RIM, neither were as large as the effect of PC1. Compared with MI, TB weakly predicted lower trust and higher fact-checking. (Because the effects of MI and EMI were similar, we refer to them interchangeably in the Discussion section as ‘market integration’; see Figure 6.) AICc model selection consistently suggested across imputations that PC1 models outperformed the other models, including the MI, EMI, TB, depend and confirmatory models (Table S5). Market integration nevertheless appeared to have a large impact on trust, compared with adherence to traditional beliefs and values. See Supporting Information for a more detailed discussion.

## Discussion

In a preregistered vignette-based experiment, we tested the roles of learning biases (PBM) and incentives (RIM) in evaluating socially learned information about grazing conditions for livestock. The PBM predicted that, if a source of information is prestigious compared with known from personal experience to be knowledgeable, people would be (a) more likely to trust and act on the advice and (b) less likely to fact-check it first. Neither of these predictions were supported. Regardless of whether the source was prestigious vs. believed from personal experience to be generally knowledgeable, trust in socially learned information about grazing conditions was equally low in both conditions, and preferences for fact-checking were also equally high in both conditions. This lack of support was found when considering ‘strong’ and ‘weak’ versions of prestige bias (*sensu* Morin, 2016; see ‘Preregistered predictions’ and ‘Study design’ sections); we tested the weak version in PBM + RIM but did not find a statistically significant effect of prestige (Table 1). Nevertheless, 24% of participants did trust the fictional advice giver, suggesting that persons known to be knowledgeable via either their prestige or via personal experience are trusted to some extent.

The RIM predicted that resource scarce participants who are less dependent on cattle would be (a) more willing to take a risk and act on socially learned information and (b) less likely to fact-check it first. Prediction (a) was supported, and prediction (b) was not. Observational measures of resource scarcity (household food insecurity scores and need) significantly predicted higher trust in, and willingness to act on, advice about livestock. Conversely, as predicted, proxy measures of livestock dependence for subsistence and household wealth predicted lower trust in the same advice. These measures, however, did not significantly impact participants’ stated need to fact-check before acting on information.

The RIM outperformed the both strong and weak versions of the PBM by AICc on both trust and fact-checking (see Tables 1 and S2). These results imply that, for Maasai in this region, risks and incentives influence trust about livestock advice, whereas the effect of prestige is indistinguishable from assessments of knowledgeability based on participants’ personal experiences. More notably, trust and reliance on social learning, at least for advice about livestock movement, were generally quite low (see also Toelch, Bruce, Newson, Richerson, & Reader, 2014; Mesoudi, Chang, Murray, & Lu, 2015).

### Exploratory analysis of regional acculturation as a predictor of trust

Regional acculturation strongly predicted trust. Acculturation was the first principal component of variables reflecting market integration vs. dependence on livestock, and traditional vs. non-traditional views about polygyny, female circumcision and cattle raiding (Figure 2). The study site comprised two distinct regions separated by a small mountain, with southern, more acculturated participants living closer to densely populated towns exhibiting higher trust, and northern, less acculturated participants living on a more rural and isolated side of the mountain exhibiting lower trust (Figures 1 and 4).

To more precisely characterise acculturation, we identified clusters of variables related to material culture (MI) and ideational culture (TB). MI was a stronger predictor of trust than TB, and a model with MI alone outperformed a model with both, suggesting that MI better explained the strong positive relationship between regional acculturation and trust. Nevertheless, the model with acculturation, which reflects covariation among many variables beyond MI, had the best performance of all (see Supporting Information for AICc tables). This suggests that acculturation was irreducible to either economic or ecological accounts alone (e.g. Edgerton, 1971). Our results also suggest that acculturation has a larger influence on advice-taking than do risks and incentives.

### Material and ideational culture

Material vs. ideational theories of culture have a long history in social sciences. Materialist accounts emphasise environmental feedback and incentive structures: individuals must learn to maximise resources and behavioural patterns varying between groups correspond to different relevant features in the environment (e.g. MI, livelihood risks). If risk and uncertainty are part of a local subsistence strategy, cultural adaptations might feature heightened sensitivity to risk (Goldschmidt & Goldschmidt, 1976; Steward, 1972). East African pastoralists optimise herd size and composition (Mace, 1990; Mace & Houston, 1989; see also Næss, Bårdsen, Pedersen, & Tveraa, 2011), and pattern herd movement based on past and current payoffs (Butt, Shortridge, & WinklerPrins, 2009; see also Domjan & Burkhard, 1986).

Ideational accounts, in contrast, emphasise beliefs, attitudes and values. Socially transmitted information can establish complex behavioural conventions (Boyd & Richerson, 1985; Tennie, Call, & Tomasello, 2009), and acculturation can be driven, at least in part, by novel ideational changes such as religious conversions or Westernisation. In Monduli Juu, missionaries fund organisations, led by Maasai locals, that advocate helping women and children gain access to formal education. Efforts to convert Maasai to Christianity have largely succeeded, in part, by appealing to women (Hodgson, 2005) and prioritising compatibility with some (but not all) Maasai traditions (Rigby, 1989). More broadly, Maasai in Monduli are well aware that their culture is shifting as a consequence of globalisation, and many anticipate that adopting new ideas will improve their lives (see also Hodgson, 1999). In our data, ideational variables covaried with materialist ones (Figure 2).

### Conflict and coordination by region

Land conflicts over grazing are a primary cause of neighbour conflicts across the broader Monduli Juu region, and large sisal plants now fence many property lines. This increases resource scarcity (e.g. available grass) and conflicts of interest among herders. Payoffs to individual vs. social learning strongly depend on the accuracy of learning (McElreath, 2004), and when misinformation is incentivised, the accuracy of social learning is reduced, and thus so is trust.

Regional variation in trust might reflect different culturally evolved solutions to a coordination problem (Binmore, 2011; Yamagishi & Suzuki, 2009), which is mutually compatible with materialist and ideational accounts. Evidence for this would include low variation within regions, and sharp discontinuities between regions (Efferson, Vogt, Elhadi, Ahmed, & Fehr, 2015; Mackie, 1996). Our data are partially consistent with this: only 8% of participants in the north trusted livestock advice compared with 51% in the south.

Based on the RIM, which was partially supported, herders should be sceptical about possibly deceptive advice about their grazing routines (e.g. Trouche et al., 2018). This is what we observe in the less market-integrated, more cattle-dependent northern region. Kinship is an important criteria for trust among Maasai (Fratkin, 2001; Spencer, 1965), and northern herders might generally mistrust non-kin with livestock advice – regardless of prestige or experience. The advice-giver in the vignette was not specified to be kin (if participants asked, they were told he was not kin).

In the south, however, trust outcomes were more split. Southern herders must routinely trust non-kin and distant relatives to successfully participate in markets. This is a novel coordination problem, because cash markets and fewer livestock also reduce the scope for land conflict among herders (see also Cronk & Leech, 2013). Market-integrated southern herders might therefore see a demand for ‘market norms’, e.g. expectations for fairness beyond kin groups (Henrich et al., 2010), which can be transmitted socially (Richerson & Boyd, 2005) or preferentially attended to by content biases (Cronk, 2017). This account was particularly well supported by regional variation in trust outcomes. Controlling for region, individual incentives did not predict additional variation in trust, possibly supporting group-level social learning processes. (Although, as noted here and in Figure 2, these incentive variables were confounded with region.)

Mistrustful southerners might reflect the recent and ongoing nature of market expansion, infrastructure development and formal education (Hodgson, 1999; Swebe, 1984). Multiple small-scale societies, including a separate Maasai community near Monduli Juu (Baird & Gray, 2014), saw disruptions in traditional social conventions after market expansion (e.g. Ensminger, 1992; Gurven, Jaeggi, von Rueden, Hooper, & Kaplan, 2015; Kasper & Borgerhoff Mulder, 2015; North, 1990). Higher livelihood diversification and lower dependence on cattle could motivate some southern herders to take strategic risks with their livestock, but cattle remain common among southern herders. This alone might explain split trust outcomes, which we did not see in the north. Alternatively, it is difficult to overstate the importance of cattle to Maasai culture, regardless of actual subsistence strategy used (Spear & Waller, 1993). It is therefore possible that these split trust outcomes near town result from risk aversion, not to livelihood risk *per se*, but to risk to cultural valuation of cattle (see also Herskovits, 1926; cf. Dahl & Hjort, 1976).

### Limitations

This study involved testing preregistered hypotheses using both experimental and observational study designs. Only one of the preregistered hypotheses regarding the RIM was supported, with observational data. Compared with experimental studies, observational studies provide weak evidence for causality, but allow researchers to study real-world behaviours that experimental studies usually cannot (e.g. Hutchins, 2000). Evidence supporting the RIM is therefore suggestive, and results should be interpreted with caution. Our vignettes also did not include a condition in which the advice giver was depicted as unknowledgeable, so we cannot determine if knowledgeability, inferred from either prestige or personal experience, influences trust. It is also worth noting that our study investigated trust in a single domain, namely, advice relating to livestock. Whether or not the findings in this study generalise to trust in other domains, such as farming, medicine or conflict resolution, is an open question.

Although we found clear evidence that acculturation was associated with trust outcomes, this key finding was not from the preregistered hypotheses but from *post hoc* exploratory analyses. Exploratory analyses are especially vulnerable to misinterpreting noise as genuine signals. Also, data in the northern vs. southern regions were collected by different research assistants, raising the possibility that regional differences in acculturation and trust were somehow a consequence of the procedures followed by each assistant. Although we cannot completely rule out an interviewer effect, we doubt it for the following reasons: both assistants were local adult men with many years of experience administering surveys. One assistant and A.D.L. separately collected data in the southern region, and their results were quite similar (i.e. a term for interviewer in regression models of data only from the southern region was not statistically significant; see the Supporting Information). Further, many of the survey items were relatively objective questions involving roof material, solar panels, number of wives, household size and so forth, where interviewer effects would not be expected, and these also differed systematically by region (see Supporting Information for tests of differences by region).

## Conclusion

Socially learned information can imply non-trivial costs and benefits, including risks of misinformation. Risk and incentives predicted increased willingness to trust in advice, but prestige did not increase trust compared with knowledgeability learned from personal experience. Acculturation, which varied markedly by region, was found to have an even larger positive association with trust. Much of this effect was due to the positive effect of market integration on trust, but weaker adherence to traditional Maasai values was also positively associated with trust to some degree. The causal pathways among market integration, acculturation and trust remain to be clarified.

## Data Availability

All preregistration materials are publicly available at https://osf.io/5p7ut. Data are available at https://doi.org/10.5281/zenodo.4118454, and supplementary information and analysis scripts are available at https://doi.org/10.5281/zenodo.4118474.

## Supplementary material

For supplementary material accompanying this paper visit https://doi.org/10.1017/ehs.2021.10. click here to view supplementary material
