# Supplementary material for: Acculturation and market integration are associated with greater trust among Tanzanian Maasai pastoralists
Source: Evol Hum Sci. 2021 Feb 10;3:e15. doi: 10.1017/ehs.2021.10 (PMC10427282; doi:10.1017/ehs.2021.10)
Supplement: Supplementary file 1 [file S2513843X21000104sup001.pdf]

Supplementary information for *Acculturation and market integration are associated with greater trust among Tanzanian Maasai pastoralists*

Aaron Lightner

2020-10-20

1 Freelists on *nkanyit* as a prestige concept

Semi-structured and key informant interviews described a person with high levels of *nkanyit* as an honorable and prudent elder who leads his community by example, caring for his many cattle, wives, and children. Composite salience scores (S) were computed by normalizing the total weighted salience (WS).

Weighted salience of each item i, mentioned by a given participant j, was computed as $WS_{i,j} = \sum_i^k \frac{r_i}{k}$, where r_i is the inverted rank of the item listed and k is the number of items listed by participant j. Note that to simplify the notation, participant j is not specified in the right-hand side of the equation, though $WS_{i,j}$ was computed per item per participant.

Composite salience was then determined by normalizing total weighted salience across participants, such that $S = \sum_j^n \frac{WS_j}{n}$. Here, n is the total sample size of the freelisting interview sample ($n = 57$). Thus, composite salience S reflects a statistic relating to how high-ranking (salient) *and* how frequently mentioned a given item is across freelists in an interviewed sample. See Quinlan (2018) for more information with examples.

Composite salience scores showed that the most important contributors to gaining nkanyit include, in descending order of importance, large cattle numbers (0.52), having and caring for a large family (0.46), being respectful to others (0.25), having good moral character (0.14), being helpful to others (0.13), and being knowledgeable, e.g., by giving good advice, being educated, and/or being intelligent (0.12). See table S1 for a full table.

2 Complete vignette text

The following vignette texts were used in our structured interviews, initially by A.D.L. with the assistance of a Maasai translator, who was either assistant 1 (from the southern area) or, in some cases, assistant 2 (from the northern area).

2.1 Prestige condition

Suppose that you are speaking with another person (*anya lomon*[1]), who is also from the Eluwai community. This person tells you about a place outside of the village, about a day's walk from here, where you should

[1]This is sometimes translated to English speakers as "exchanging news", but the literal Maa translation is "eating words". *Anya lomon* appears to be compulsive and frequent, somewhat ritualistic, and follows a consistent question-answer format with a heavy use of phatic sounds from the listener. It is therefore easy for participants to imagine this type of scenario, as it refers to a common and important method for staying informed on a daily basis.

Table S1: Composite salience scores for freelisted domains, mentioned in response to a interview questions about how a person gains nkanyit.

coded domain	composite salience
has cattle or wealth	0.52
family	0.46
gives respect	0.25
has good character	0.14
helps others	0.13
has knowledge or education	0.12
religious	0.05
good standing in community	0.05
is an elder	0.03
resolves conflicts	0.02

take your livestock for grazing because there is plenty of grass and water available over there. This person advising you is a person you know, because he is someone in your community who has a lot of *nkanyit*.

(On a scale of 1-10)[2], how much do you believe this person? (*trust outcomes*)
If you were considering following this person's advice, would you need to travel there yourself to see if they were telling the truth? (*fact-checking outcomes*)

2.2 Experience condition

Suppose that you are speaking with another person (*anya lomon*), who is also from the Eluwai community. This person tells you about a place outside of the village, about a day's walk from here, where you should take your livestock for grazing because there is plenty of grass and water available over there. This person advising you is a person you know, because he is someone you have known from personal experience to be very knowledgeable.

(On a scale of 1-10), how much do you believe this person? (*trust outcomes*)
If you were considering following this person's advice, would you need to travel there yourself to see if they were telling the truth? (*fact-checking outcomes*)

3 Coding our outcome variable

Trust outcomes were coded on a three-point scale (1 = completely trust, 0.5 = somewhat trust, 0 = does not trust). Fact-checking outcomes were measured as simple yes/no responses (1 = yes, 0 = no).

Coding trust outcomes onto a three-point scale was motivated strictly by a challenge in the data collection process, and we documented this prior to analyzing data in our preregistration osf.io/5p7ut. Trust outcomes were initially, for most interviews conducted by A.D.L., on a scale of 1-10. Most participants found scales of 1-10 very unintuitive, so A.D.L. used a carefully measured visual aid on cardstock, allowing participants to point to a location on the scale.

Participants, however, found this visual scale to be much more intuitive when A.D.L. evoked three salient reference points: left means no trust at all, middle means some trust, and right means complete trust. Many participants had ignored the scale completely and simply answered "yes, completely" or "no, not at all". The two local assistants framed the same question by exclusively using these three salient reference points as

[2]This was mainly used by A.D.L., but was abandoned during most of the interviews conducted by the local research assistants. Coded trust outcomes were established prior to entering or analyzing data, based on our experiences communicating this scale to participants. See the following section for more information.

Table S2: Model comparison of logistic regression models used in our confirmatory analyses, using AICc scores and weights as our selection criteria to compare models with trust outcomes (left) and fact-checking outcomes (right).

	trust models	K	AICc	Delta_AICc	ModelLik	AICcWt	LL	Cum.Wt
2	RIM	5	198.85	0.00	1.00	0.44	-94.26	0.44
3	PBM+RIM	6	199.34	0.49	0.78	0.34	-93.44	0.78
1	PBM	2	200.19	1.34	0.51	0.22	-98.06	1.00

check models	K	AICc	Delta_AICc	ModelLik	AICcWt	LL	Cum.Wt
PBM	2	171.65	0.00	1.00	0.56	-83.79	0.56
RIM	5	173.22	1.57	0.46	0.26	-81.45	0.82
PBM+RIM	6	173.96	2.31	0.31	0.18	-80.75	1.00

options, asking participants if they had complete trust, some trust, or no trust in the advice given (which they recorded as 10, 5, and 1, respectively).

It therefore made sense to code responses onto a three-point scale, because it not only more accurately reflects the data collection process used by each interviewer, but also the way that most participants interpreted the question about "how much" they trusted the advice. Responses on a scale of 1-10 appeared to be routinely thought about with respect to their closeness/distance to/from 1, 5, and 10 in interviews with A.D.L. The three-point scale we coded responses onto were 0, 0.5, 1, and responses to A.D.L. were converted by dividing the 1-10 scale into increments of 3, according to the following rule: $i < 4 \rightarrow 0$, $4 \leq i < 7 \rightarrow 0.5$, and $i \leq 7 \rightarrow 1$.

In effect, this means that for the participants interviewed by A.D.L., people who pointed closest to the middle of the line were assigned the middle value on a 3-point scale, whereas people who pointed closest to one of the extremes were assigned their corresponding values on that same scale. More straightforwardly, responses collected by the two local research assistants were converted as 1 (not at all trusting) was assigned to 0, 5 (somewhat trusting) assigned to 0.5, and 10 (completely trusting) assigned to 1.

Although this decision was based solely on constraints on our data collection method, we investigate the question of if and how this might have substantially affected our results in a section below. (It did not, as we will show in the following sections.)

4 Confirmatory analyses

In the main article text, under Confirmatory analyses (Results section), we included a single effects plot showing our supported predictions for trust outcomes in the RIM. Here, we include effects plots from the PBM (figure S1) and fact-checking outcomes for the RIM (figure S2), which did not show a statistically significant effect conforming to our predictions.

4.1 AICc model selection

For trust outcomes, model selection using weighted AICc showed that the RIM had better performance than the PBM and the PBM+RIM. For fact-checking outcomes, PBM had slightly better performance than the RIM and PBM+RIM, although it is worth emphasizing: *none* of these models showed a statistically significant effect for fact-checking outcomes, and a larger number of parameters in RIM accounts for its underperformance here. Furthermore, model comparisons in our confirmatory analysis, while conforming to our preregistration, involves only 216 out of 225 observations in each model, after complete cases. We therefore re-evaluate the PBM, RIM, and PBM+RIM in the exploratory analyses below, using multiple imputation to make use of the full dataset. See table S2.

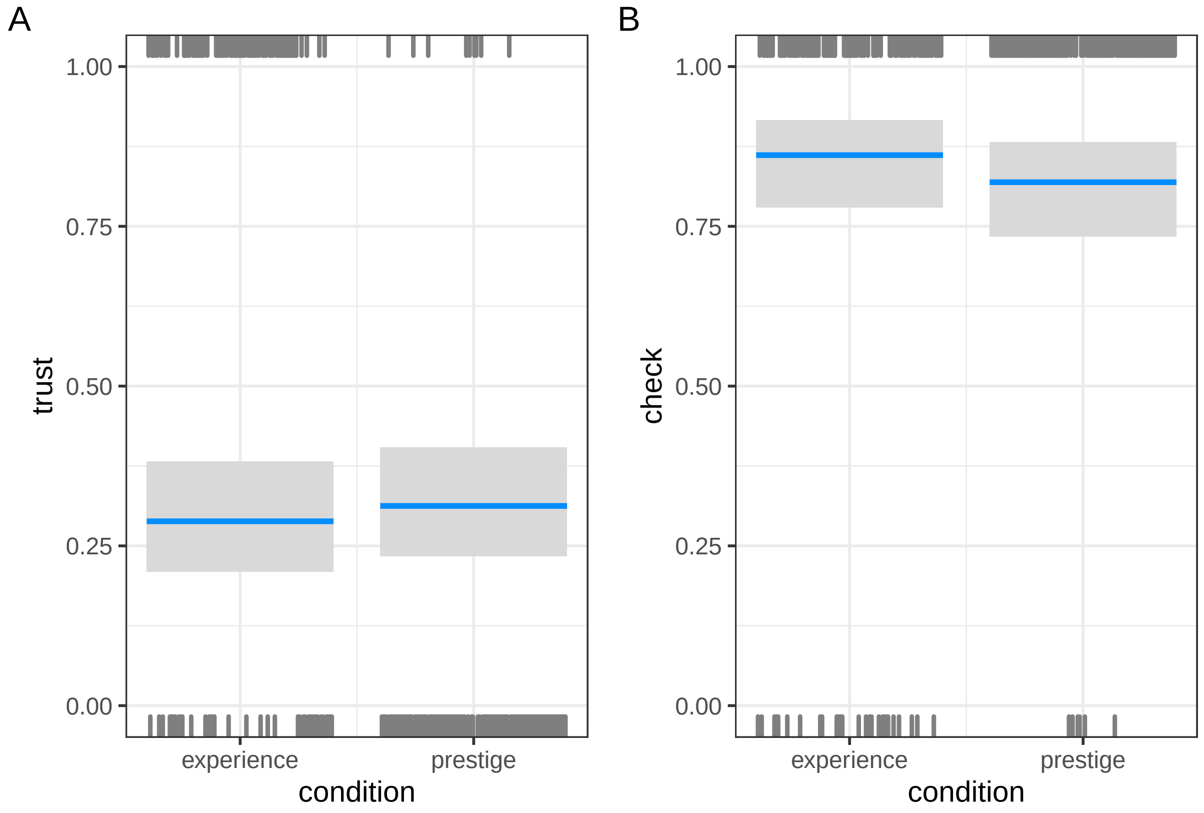

Figure S1: Logistic regression model for PBM predictors on trust outcomes (A) and fact-checking outcomes (B). Model coefficients are shown in table 2 (columns 1 and 4) of the main article.

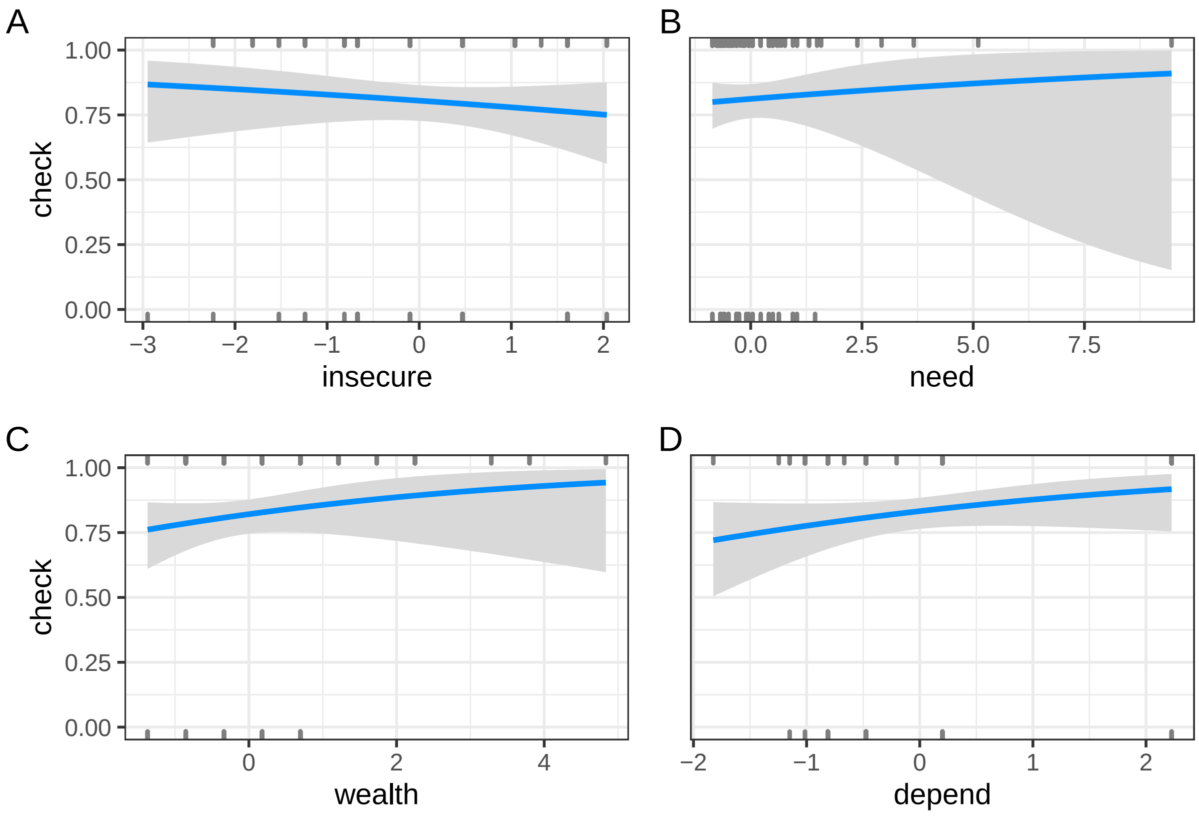

Figure S2: Logistic regression models for RIM predictors on fact-checking outcomes. Model coefficients are in table 2 (column 5) of the main article.

4.2 Re-analyzing confirmatory predictions after questioning our decisions

To stay consistent with our preregistration, we (1) used logistic regression on proportional outcomes, rather than ordered logistic regression on our three-point scale, and (2) transformed trust outcomes into that three-point scale, based on confusion among participants about judging on scales of 1-10. Here, we re-analyze the data to address the question of if and how either of these decisions might have affected the results on our trust outcomes.

4.2.1 Did logistic regression on proportional trust outcomes affect the results?

First, we re-ran trust models using an ordered logistic regression and found similar effects in each of our models in the main text, which, based on our preregistration, used logistic regression on proportional outcomes. In other words, analyzing our data using logistic regression on proportional outcomes (which we did in the main text) vs. ordered logistic regression (which now do here) did not substantially change our results in the confirmatory analyses, nor in the exploratory analyses. See table S3, and figures S3 and S4 for results of the ordered logistic regression based on ranked categorical responses.

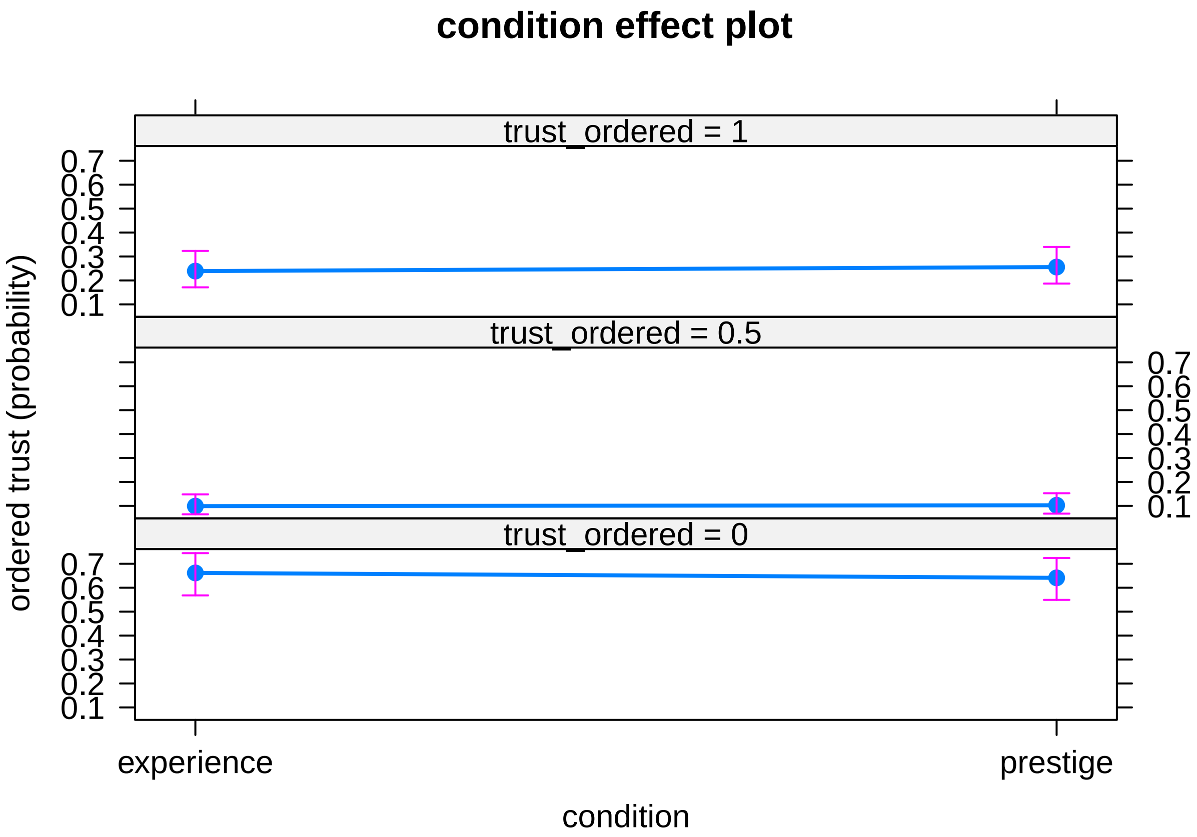

Figure S3: Effects plot for PBM using ordered logistic regression with trust outcomes on a categorical three-point scale.

4.2.2 Did coding trust outcomes onto a three-point scale affect the results?

Second, we re-ran trust models using the ten-point scale that some participants initially tried to respond with, when A.D.L. was present to explain it to them. Trust outcomes based our initial data collection (i.e., some

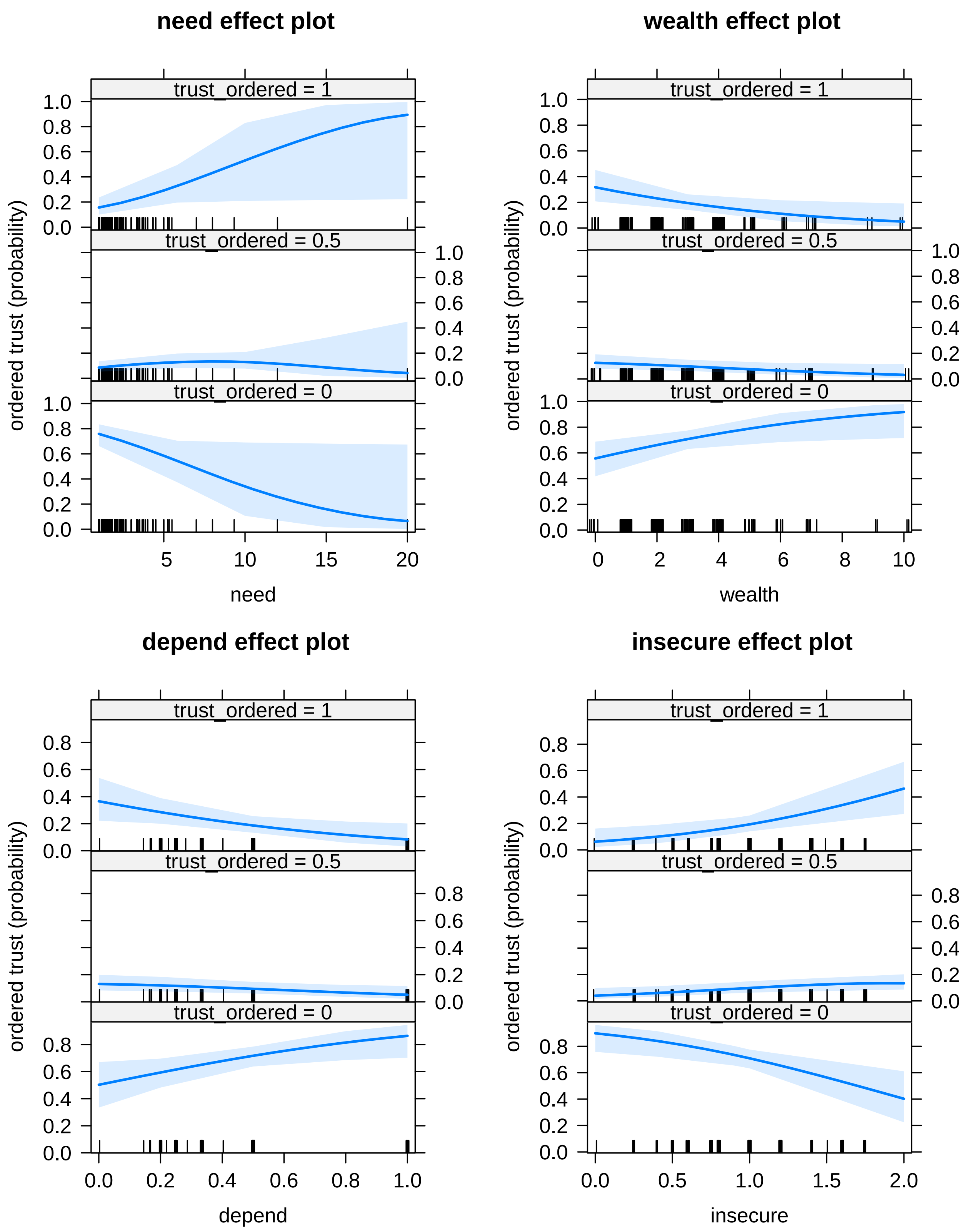

Figure S4: Effects plot for RIM using ordered logistic regression with trust outcomes on a categorical three-point scale.

Table S3: Ordered logistic regression models for trust outcomes (on an ordered three-point scale), based on condition (PBM, column 1), and on scaled measures of household food insecurity, need, wealth, and dependence on livestock as a source of subsistence (RIM, column 2).

	Dependent variable:	
	trust_ordered	
	(1)	(2)
conditionprestige	0.090	
	(0.279)	
need		0.201*
		(0.100)
wealth		−0.219*
		(0.097)
depend		−1.840*
		(0.792)
insecure		1.285**
		(0.449)
Note:	*p<0.05; **p<0.01; ***p<0.001	

participants attempting to respond on a ten-point scale, but preferring the more intuitive three-point scale[3]) only involved recoding of 18% of all data points (as described here in sect. 3). The ten-point scale outcomes were strongly correlated with those in our coded three-point scale, which were used in our main results ($r = 0.98$, $p = 6 \times 10^{-155}$). Our re-analysis shows that the inclusion of the ten-point scale responses largely does not affect our results, although in the RIM, effects of our proxy measures of household wealth and need are slightly weakened in particular. See figures S5 and S6 for effects plots, and table S4 for regression coefficients and statistics.

5 Regional variation in responses

Responses in PC1 (figure 2, main article) appeared to be a regional acculturation variable that was low in the northern region and high in the southern region. Similarly, responses in the northern vs. southern regions varied on trust outcomes (north: 1 = 51%, 0.5 = 24%, 0 = 25%; south: 1 = 7.8%, 0.5 = 1.4%, 0 = 86%) and fact-checking outcomes (north: 1 = 69%, 0 = 31%; south: 1 = 92%, 0 = 8%). For a mosaic plot visualizing this large regional disparity, see figure S7.

5.1 Regional differences vs. interviewer differences

As discussed in our limitations (see Discussion section in the main text), it is possible that northern vs. southern regions were somehow a consequence of different interviewers, rather than of true regional differences. As we also claim in the main text, however, we doubt this for at least two reasons.

First, A.D.L. and assistant 1 separately collected data in the southern region, and their results within this region were similar overall. Second, important regional differences, which were included in our PC1

[3]See section 3 in this document for a detailed description of the original measurement methods and how the ten-point scale was coded into the three-point scale for trust outcomes.

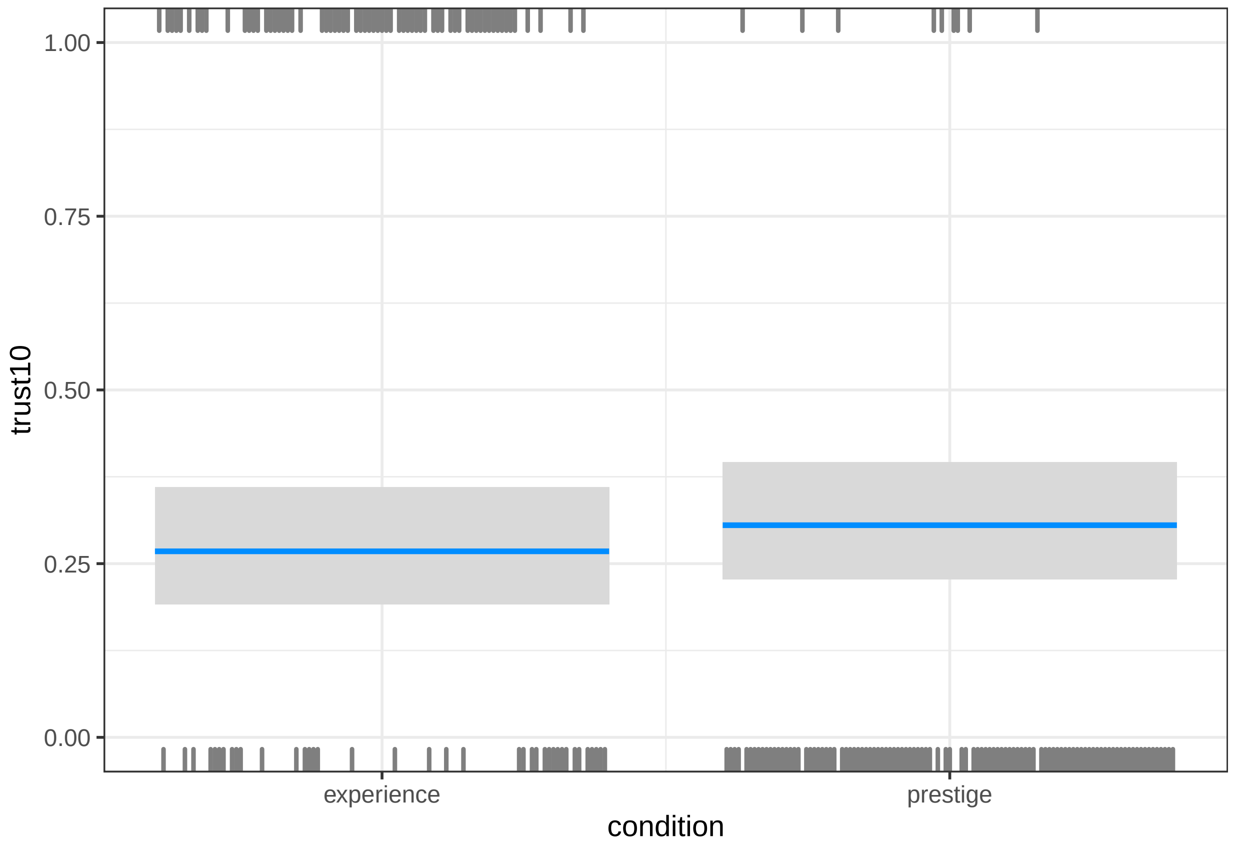

Figure S5: Effects plot for PBM using logistic regression with trust outcomes, using our initial use of a ten-point scale for trust outcomes (prior to coding onto three-point scale).

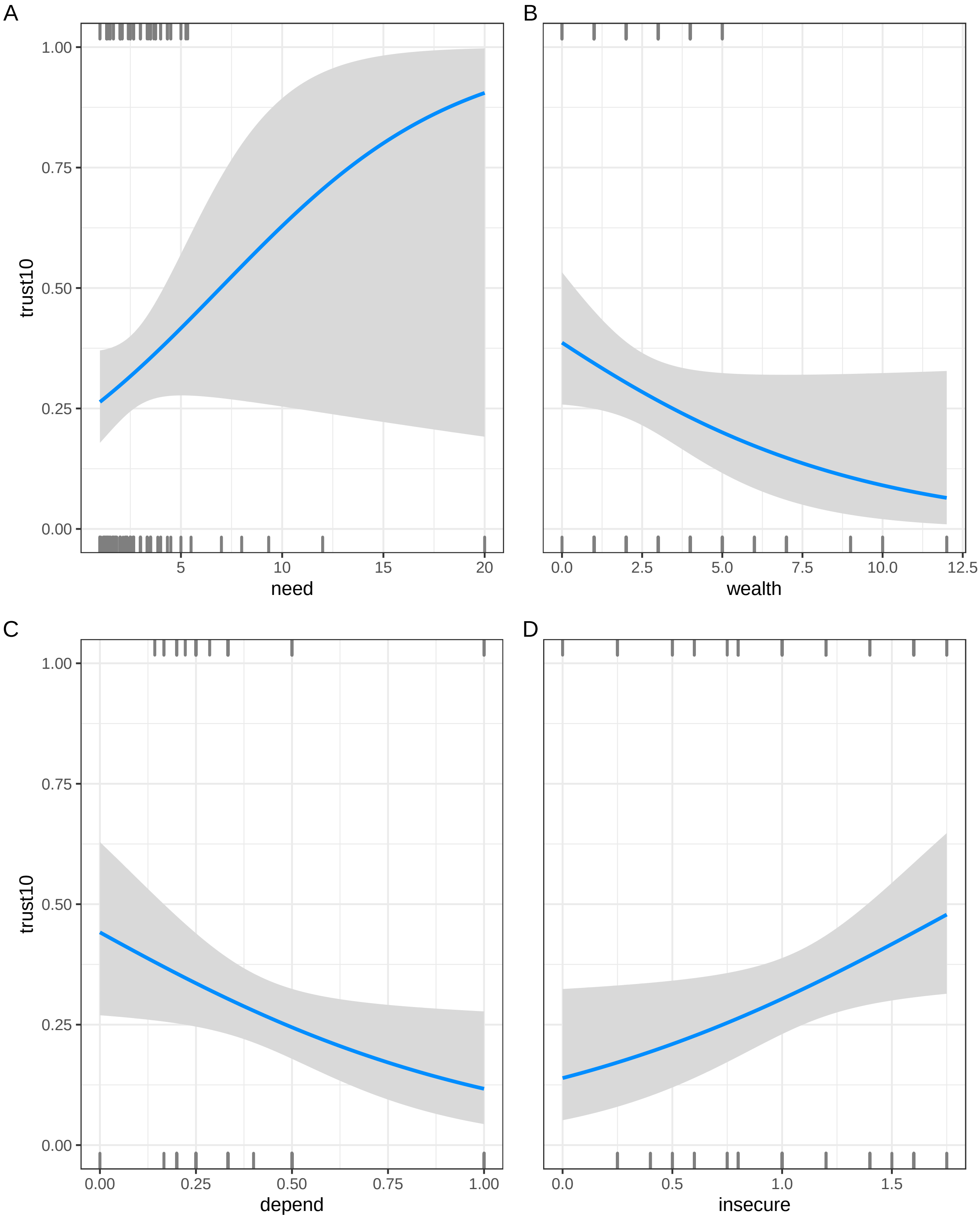

Figure S6: Effects plot for RIM using logistic regression with trust outcomes, using our initial use of a ten-point scale for trust outcomes (prior to coding onto three-point scale).

Table S4: Logistic regression models for trust outcomes, including the ten-point scale used unsuccessfully in some our sample, based on condition (PBM, column 1), and on scaled measures of household food insecurity, need, wealth, and dependence on livestock as a source of subsistence (RIM, column 2).

	Dependent variable:	
	trust10	
	(1)	(2)
conditionprestige	0.184	
	(0.301)	
need		0.173
		(0.108)
wealth		−0.185
		(0.101)
depend		-1.787^{*}
		(0.855)
insecure		0.992^{*}
		(0.472)
Constant	-1.006^{***}	−1.228
	(0.220)	(0.755)
Note:	*p<0.05; **p<0.01; ***p<0.001	

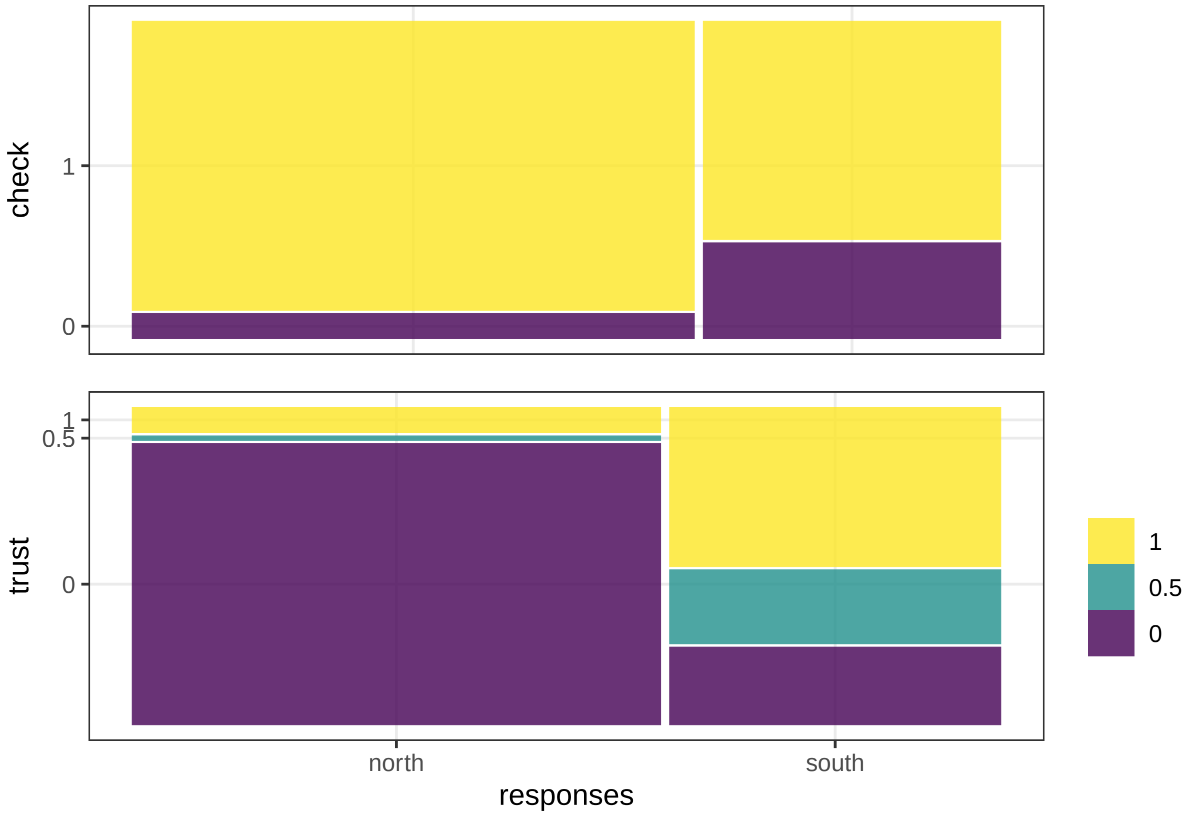

Figure S7: Mosaic plot showing response counts for each trust outcomes (lower) and fact-checking outcomes (upper) by region (colors).

acculturation variable, also included relatively straightforward and objective survey items that were unlikely to result from an interviewer effect. These included roof material, solar panels, and number of wives.[4] We address each of these two claims here.

5.1.1 Including an interviewer term in our southern regression models

Within our southern region data, we do not find a substantial interviewer effect on trust outcomes (figure S8) and fact-checking outcomes (figure S9). We also do not generally find interviewer effects in the southern region data when including an interviewer term in the confirmatory and exploratory models, although fact-checking outcomes might be a slight exception in some cases – see table S5. Overall, we do not find a strong interviewer effect on trust and fact-checking outcomes in the southern region, suggesting that data were not collected differently by A.D.L. and interviewer 1.

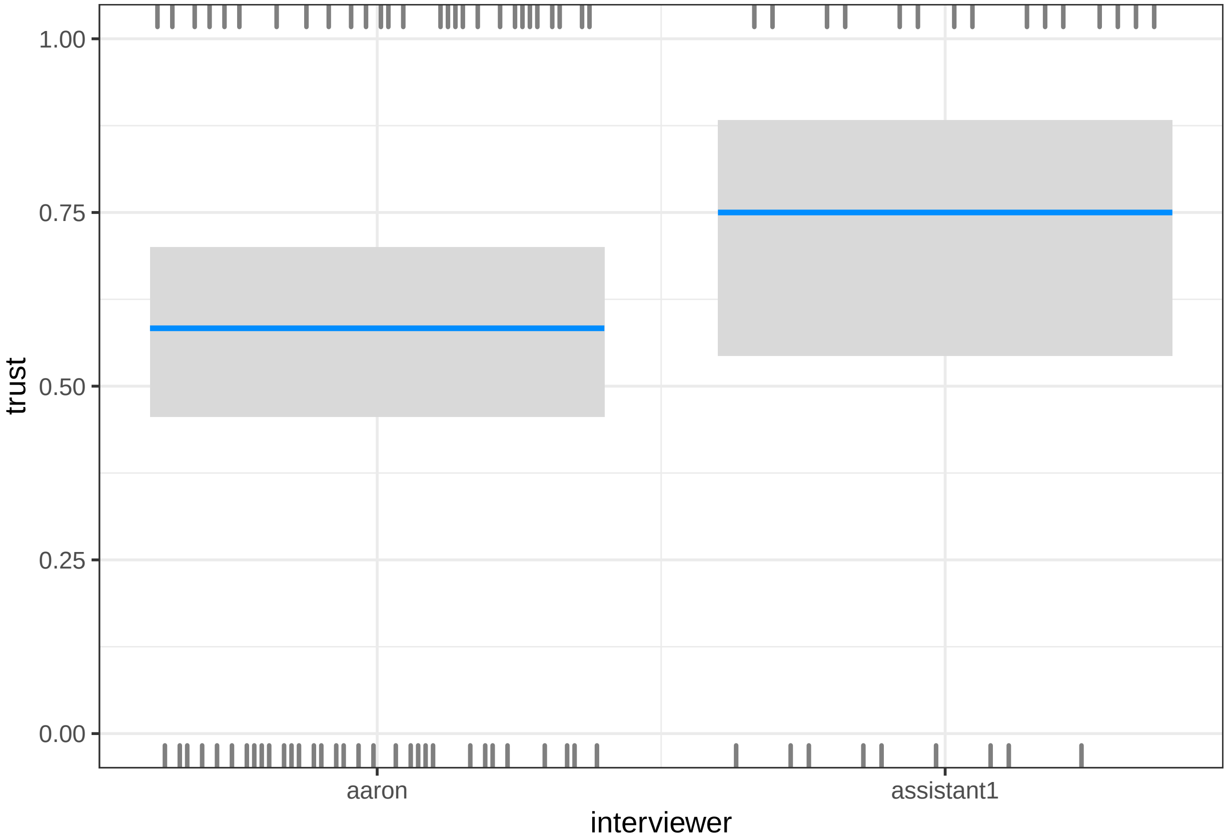

Figure S8: Effects plot using logistic regression to model trust outcomes as a function of interviewer in the southern region.

[4]It is also worth emphasizing that interviewers 1 and 2 are both highly experienced in administering scientific research, and are both local and respected adults. Each interviewer was trained directly by A.D.L. in the survey, communicated with him when they had questions, and practiced administering the survey by translating for A.D.L. prior to administering the survey independently.

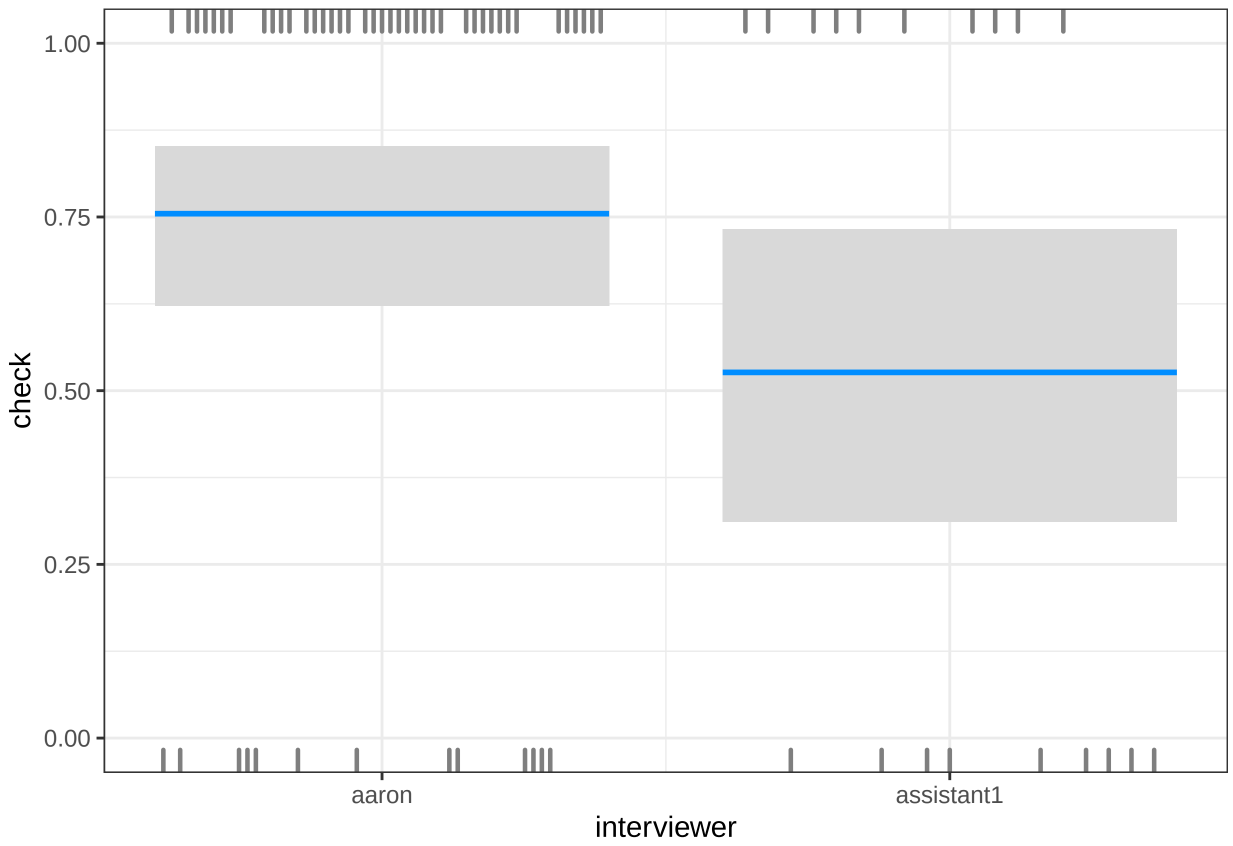

Figure S9: Effects plot using logistic regression to model fact-checking outcomes as a function of interviewer in the southern region.

Table S5: Logistic regression models for trust outcomes and fact-checking outcomes in the southern region, with interviewer term included in each model. Columns 1 and 6 correspond to the effects plots in figures S8 and S9, and the remaining columns correspond to our confirmatory results (PBM, RIM, PBM+RIM) and key exploratory result (PC1).

	Dependent variable:									
	trust					check				
	(1)	(2)	(3)	(4)	(5)	(6)	(7)	(8)	(9)	(10)
conditionprestige		−0.40		−0.36			−0.19		−0.42	
		(0.47)		(0.52)			(0.53)		(0.61)	
insecure			0.15	0.12				−0.01	−0.03	
			(0.24)	(0.25)				(0.29)	(0.29)	
need			0.04	0.04				0.75	0.80	
			(0.25)	(0.25)				(0.60)	(0.62)	
wealth			−0.30	−0.31				−0.06	−0.09	
			(0.25)	(0.25)				(0.30)	(0.30)	
depend			0.08	0.11				−0.002	0.04	
			(0.28)	(0.28)				(0.39)	(0.39)	
pc1					0.20					−0.13
					(0.44)					(0.51)
interviewerassistant1	0.76	0.77	0.57	0.56	0.82	−1.02	−1.03	−0.79	−0.85	−1.05
	(0.54)	(0.54)	(0.58)	(0.58)	(0.55)	(0.56)	(0.56)	(0.62)	(0.63)	(0.57)
Note:								*p<0.05; **p<0.01; ***p<0.001		

5.1.2 Consistent regional differences on straightforward and objective measures

A remaining test for a possible interviewer effect is whether or not the most straightforward and objective observational data also vary by region. The key here is to analyze measures that are not likely subject to interviewer effects. Suppose, for example, that trust outcomes vary by region (which they do, as shown in figure S7), but measures requiring little-to-no participant input do not. This would be consistent with the idea that response variation was a result of different interviewers. Now suppose, in contrast, the regional differences that are easy to measure and do not likely involve interviewer effects *also* vary by region. This would be consistent with the idea that these differences, like other variables in PC1 (acculturation), result from true regional differences. Here, we consider three observational measures that are extremely unlikely to result from interviewer effects: presence/absence of a metal roof, presence/absence of a solar panel, and number of wives in the household.

When comparing differences in roof material by region, an especially stark and plainly observable difference by region is in roof material, a reliable proxy measure for cash wealth and market access. The proportion of southern participants owning a metal roof is 40%, in contrast to the 0% of northern participants owning a metal roof. Crucially, this is both unsurprising and consistent with our key findings in the main text: metal roof construction does not only require cash and access to purchased materials in town, but also requires sufficient infrastructure (i.e., road access) to transport the materials to a household for construction. As A.D.L. observed during fieldwork, transporting such materials is challenging but doable in the southern region, but virtually impossible in the northern region.

Similarly, we see a higher proportion of solar panel ownership among southern participants, which was 41%, in contrast to the 16% among northern participants (Fisher's exact test: $OR = 3.6$, $p = 8.5 \times 10^{-5}$). This regional trend is consistent with our key findings in the main text because solar panel ownership is another useful proxy indicator of cash wealth: not only are they purchased, but as key informants mentioned, they usually involve monthly (cash) payments to a rental company that owns the panel. These are typically installed on the (metal or grass) roof, and are not constrained by transportation requirements like metal roofs are. Lastly, in the more traditional/less market integrated northern region, we also saw more wives per household (north: 2.6, south: 1.8; Wilcoxon rank sum test: $W = 7009.5$, $p = 0.0015$), which is also consistent with the key results in our exploratory analyses.

These trends are each consistent with the main findings of our study, and are much less likely to result from interviewer differences than from regional differences in market access, cash wealth, and possibly broader social and cultural differences (which we discuss further in the main text; see Discussion section).

6 Exploratory analyses with multiple imputation

Exploratory analyses used the mice package (van Buuren 2020) to conduct multiple imputation, pooling results from five imputed datasets. Here, we show a walkthrough of variable selection and quality checks on the multiple imputed datasets. This section includes a follow-up on our confirmatory analyses, which we included in the exploratory analyses after imputation, finding similar results to those in our preregistered confirmatory analysis. We show our selection procedure for variable inclusion here.[5] After selecting our quantitative variables for inclusion (53 variables), we were left with a remaining dataset with 1.8% of all observations missing.

6.1 Selecting variables for inclusion

Many questions in our survey contained missing data. Some questions contained very large amounts of missing data, particularly on certain items for which A.D.L. needed to be present (e.g., to guide follow up questions). All quantitative variables in our dataset were initially considered candidates for inclusion in our

[5]Note that our final sample used in the exploratory analyses, *after* multiple imputation, was 216 observations, because we did not impute outcomes variables (which each had a few missing cases)

exploratory analyses, which involved PCA and model comparisons. Both of these analyses required complete cases, which we addressed with multiple imputation (see details in the next section). We first needed to select a subset of our candidate variables missing only a few observations, along with a non-arbitrary way of defining “a few”. As an initial heuristic, we considered $< 10\%$ missing data per column (about 23 missing observations, maximum) to be ideal.

Plotting the number of missing observations per candidate variable, we looked for a large gap in number of missing observations that might suggest a low cutoff, roughly optimizing our tradeoff between maximizing variable inclusion and minimizing numbers of missing observations. See figure S10. Notice two things about this figure. First, variable names along the y-axis are not relevant to our decision process to include vs. exclude, so they are not labeled here (if anything, knowing variable names here would have possibly biased this procedure). Second, there is a large gap on the dot chart between the blue variables and the red variables. The maximum number of missing observations in the blue variables is 10, and the next largest number of missing observations (i.e., minimum number of missing observations in the red variables) is 21. Hence, we used 10 missing observations as our threshold for inclusion in the multiple imputation.

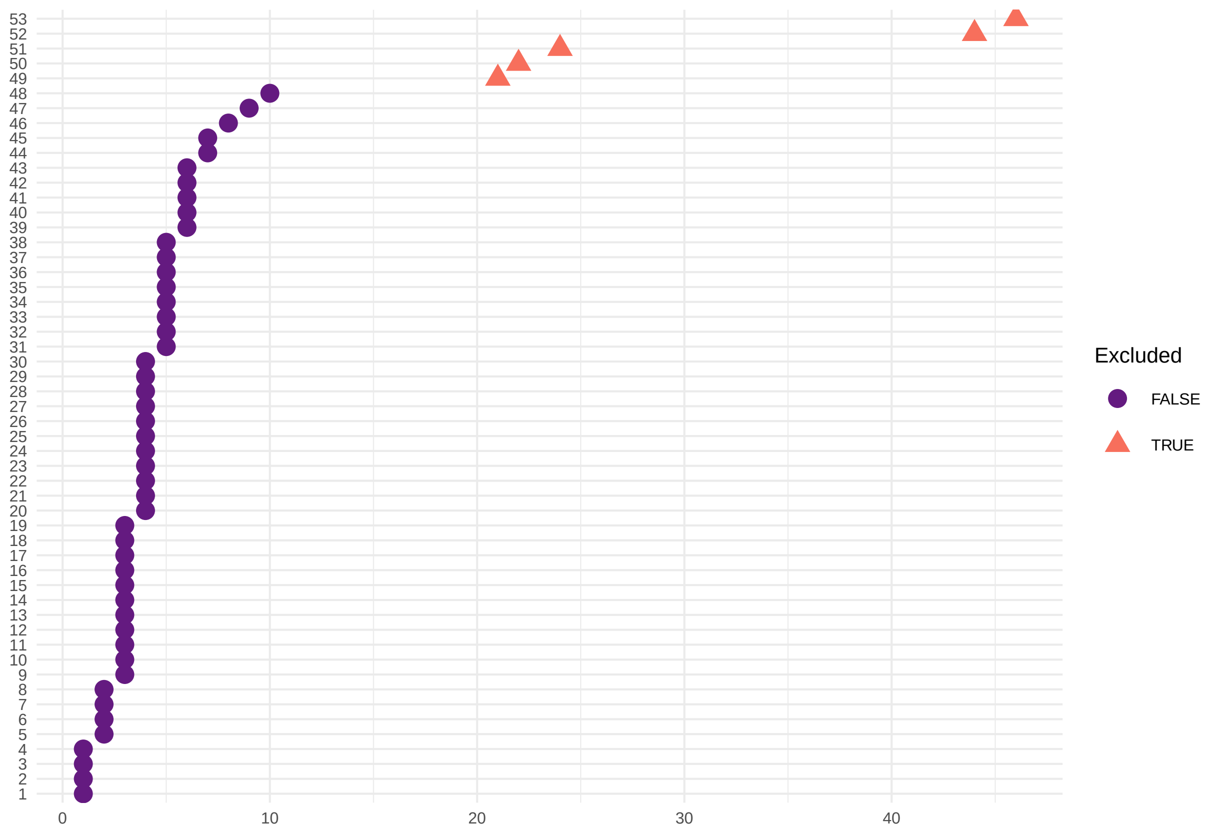

Figure S10: Dot plot showing number of missing observations (x-axis) for quantitative variables containing 1 or more missing observations, which were considered for inclusion in multiple imputation and PCA (y-axis). Blue dots correspond to variables we included, with 10 or fewer missing observations. Red dots correspond to variables we excluded, with more than 10 missing observations.

6.2 PC1 variation between imputed datasets

To check for possible variation in our PCA results on our multiple imputed datasets, we analyzed PC1 outcomes between the five imputed datasets. Specifically, we investigated the pointwise standard deviation on PC1 between datasets. (Note that these are standard deviations computed from 5 observations, which are susceptible to some noise.) See figure S11.

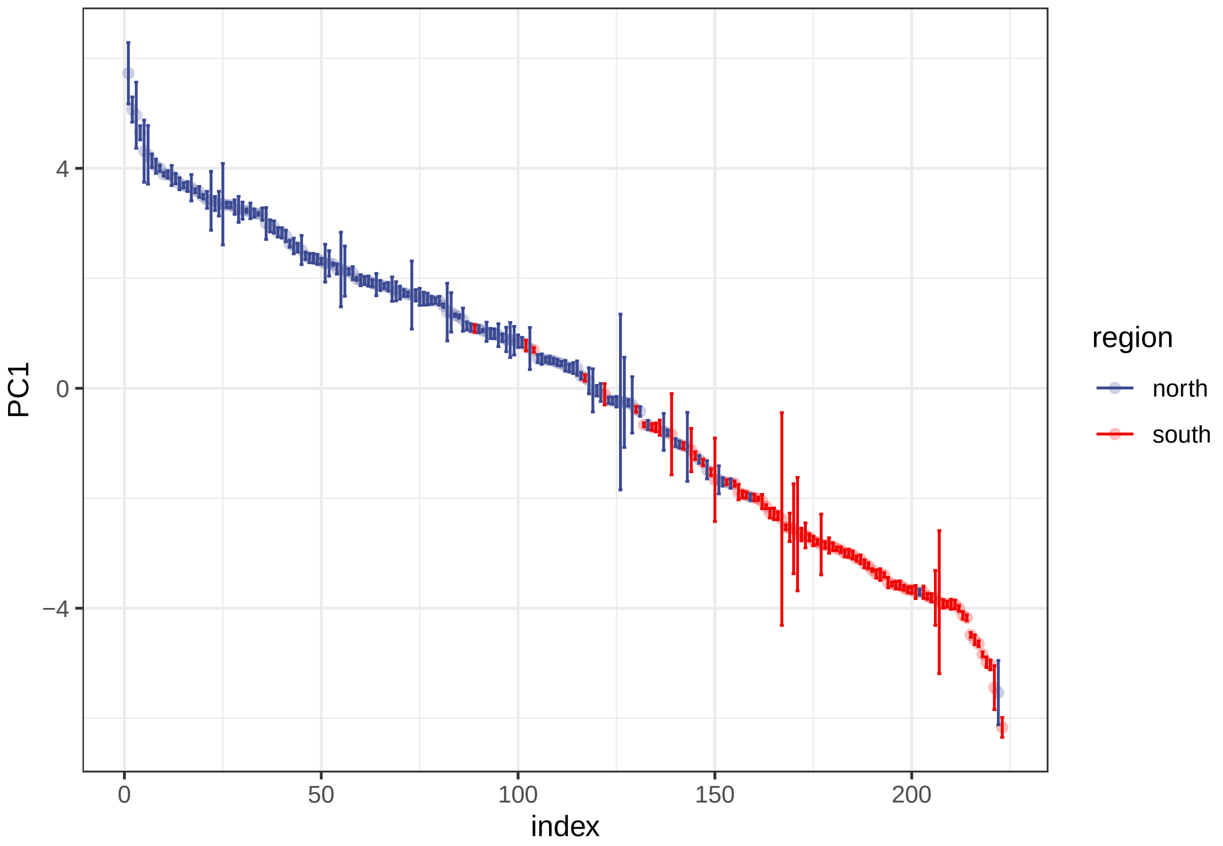

Figure S11: Ordered PC1 outcomes for each participant in five imputed datasets. Points are mean PC1 outcomes between imputations, error bars are +/- 2 standard deviations, and colors are region.

6.3 AICc tables for each imputed dataset

Results from our model selection were largely consistent across imputations, though with a few minor exceptions. See table S6 for model selection based on trust outcomes, and table S7 for model selection based on fact-checking outcomes. Note that our confirmatory results here do not substantially change after imputing the data and re-analyzing the PBM, RIM, and PBM+RIM. Recall that MI refers to our *a priori* measure of market integration, whereas EMI refers to our cluster found in the hierarchical cluster analysis discussed in the main text (e.g., figure 5 in the article). The model with acculturation (PC1), which reflects covariation among many variables beyond MI, had the best performance of all.

6.4 Model estimates before and after pooling

Each of the models in our AICc model comparison above were individually analyzed prior to pooling results. Pooled results are shown in the coefficients plot (figure 6) of the main article text, and statistics are report here (table S8). Each of these pooled results conform closely to the results from each individual imputed dataset, which we report individually here for trust and fact-checking outcomes. See tables S9-S18.

Table S6: Model comparison of logistic regression models using AICc scores and weights as our selection criteria to compare models (trust outcomes). Each refers to a separate imputed dataset.

Modnames	K	AICc	Delta_AICc	ModelLik	AICcWt	LL	Cum.Wt
pc1	2	212.48	0.00	1.00	0.56	-104.21	0.56
pc1_pbm	3	212.98	0.50	0.78	0.44	-103.43	1.00
EMI	2	226.06	13.57	0.00	0.00	-111.00	1.00
hclust	3	227.67	15.19	0.00	0.00	-110.78	1.00
MI	2	228.28	15.80	0.00	0.00	-112.11	1.00
dep	2	241.38	28.90	0.00	0.00	-118.66	1.00
ETB	2	246.47	33.99	0.00	0.00	-121.21	1.00
RIM	5	246.48	34.00	0.00	0.00	-118.10	1.00
PBM_RIM	6	247.30	34.82	0.00	0.00	-117.45	1.00
PBM	2	249.42	36.94	0.00	0.00	-122.68	1.00
pc1	2	210.94	0.00	1.00	0.56	-103.44	0.56
pc1_pbm	3	211.42	0.48	0.79	0.44	-102.65	1.00
MI	2	223.35	12.41	0.00	0.00	-109.65	1.00
EMI	2	224.34	13.40	0.00	0.00	-110.14	1.00
hclust	3	226.04	15.10	0.00	0.00	-109.96	1.00
dep	2	240.77	29.83	0.00	0.00	-118.36	1.00
RIM	5	244.97	34.03	0.00	0.00	-117.34	1.00
PBM_RIM	6	245.80	34.87	0.00	0.00	-116.70	1.00
ETB	2	246.54	35.60	0.00	0.00	-121.24	1.00
PBM	2	249.42	38.48	0.00	0.00	-122.68	1.00
pc1	2	211.01	0.00	1.00	0.57	-103.48	0.57
pc1_pbm	3	211.58	0.57	0.75	0.43	-102.73	1.00
EMI	2	225.76	14.75	0.00	0.00	-110.85	1.00
MI	2	226.30	15.29	0.00	0.00	-111.12	1.00
hclust	3	227.40	16.40	0.00	0.00	-110.65	1.00
dep	2	239.93	28.93	0.00	0.00	-117.94	1.00
RIM	5	244.62	33.61	0.00	0.00	-117.17	1.00
PBM_RIM	6	245.39	34.38	0.00	0.00	-116.49	1.00
ETB	2	246.42	35.41	0.00	0.00	-121.18	1.00
PBM	2	249.42	38.41	0.00	0.00	-122.68	1.00
pc1	2	211.31	0.00	1.00	0.56	-103.63	0.56
pc1_pbm	3	211.80	0.49	0.78	0.44	-102.84	1.00
EMI	2	225.74	14.43	0.00	0.00	-110.84	1.00
MI	2	226.86	15.55	0.00	0.00	-111.40	1.00
hclust	3	227.26	15.95	0.00	0.00	-110.58	1.00
dep	2	239.23	27.92	0.00	0.00	-117.59	1.00
RIM	5	243.29	31.98	0.00	0.00	-116.50	1.00
PBM_RIM	6	243.90	32.59	0.00	0.00	-115.75	1.00
ETB	2	246.16	34.85	0.00	0.00	-121.05	1.00
PBM	2	249.42	38.11	0.00	0.00	-122.68	1.00
pc1	2	211.41	0.00	1.00	0.56	-103.68	0.56
pc1_pbm	3	211.88	0.47	0.79	0.44	-102.88	1.00
EMI	2	225.06	13.65	0.00	0.00	-110.50	1.00
MI	2	225.73	14.32	0.00	0.00	-110.84	1.00
hclust	3	226.62	15.21	0.00	0.00	-110.25	1.00
dep	2	239.93	28.52	0.00	0.00	-117.94	1.00
RIM	5	244.70	33.30	0.00	0.00	-117.21	1.00
PBM_RIM	6	245.44	34.03	0.00	0.00	-116.52	1.00
ETB	2	246.14	34.73	0.00	0.00	-121.04	1.00
PBM	2	249.42	38.01	0.00	0.00	-122.68	1.00

Table S7: Model comparison of logistic regression models using AICc scores and weights as our selection criteria to compare models (fact-checking outcomes). Each refers to a separate imputed dataset.

Modnames	K	AICc	Delta_AICc	ModelLik	AICcWt	LL	Cum.Wt
pc1	2	173.24	0.00	1.00	0.43	-84.59	0.43
pc1_pbm	3	173.84	0.60	0.74	0.32	-83.86	0.75
MI	2	175.51	2.27	0.32	0.14	-85.72	0.89
EMI	2	177.32	4.08	0.13	0.06	-86.63	0.95
hclust	3	178.06	4.82	0.09	0.04	-85.97	0.99
dep	2	182.23	8.99	0.01	0.00	-89.09	0.99
ETB	2	182.32	9.08	0.01	0.00	-89.13	1.00
PBM	2	184.65	11.41	0.00	0.00	-90.29	1.00
RIM	5	185.21	11.97	0.00	0.00	-87.45	1.00
PBM_RIM	6	185.84	12.60	0.00	0.00	-86.71	1.00
pc1	2	173.43	0.00	1.00	0.42	-84.69	0.42
pc1_pbm	3	174.04	0.61	0.74	0.31	-83.96	0.72
MI	2	175.07	1.64	0.44	0.18	-85.51	0.91
EMI	2	177.83	4.39	0.11	0.05	-86.88	0.95
hclust	3	178.55	5.12	0.08	0.03	-86.21	0.99
dep	2	182.14	8.71	0.01	0.01	-89.04	0.99
ETB	2	182.37	8.93	0.01	0.00	-89.15	1.00
PBM	2	184.65	11.21	0.00	0.00	-90.29	1.00
RIM	5	185.25	11.81	0.00	0.00	-87.47	1.00
PBM_RIM	6	185.91	12.48	0.00	0.00	-86.74	1.00
pc1	2	173.11	0.00	1.00	0.46	-84.52	0.46
pc1_pbm	3	173.75	0.64	0.73	0.33	-83.81	0.79
MI	2	175.78	2.68	0.26	0.12	-85.86	0.91
EMI	2	177.83	4.73	0.09	0.04	-86.89	0.96
hclust	3	178.58	5.47	0.06	0.03	-86.23	0.99
dep	2	181.95	8.84	0.01	0.01	-88.94	0.99
ETB	2	182.35	9.24	0.01	0.00	-89.14	1.00
PBM	2	184.65	11.54	0.00	0.00	-90.29	1.00
RIM	5	185.20	12.09	0.00	0.00	-87.45	1.00
PBM_RIM	6	185.83	12.73	0.00	0.00	-86.71	1.00
pc1	2	173.40	0.00	1.00	0.46	-84.67	0.46
pc1_pbm	3	174.02	0.62	0.73	0.33	-83.95	0.79
MI	2	176.00	2.60	0.27	0.12	-85.97	0.91
EMI	2	178.23	4.83	0.09	0.04	-87.09	0.95
hclust	3	178.75	5.35	0.07	0.03	-86.31	0.98
ETB	2	182.09	8.69	0.01	0.01	-89.02	0.99
dep	2	182.10	8.70	0.01	0.01	-89.02	1.00
PBM	2	184.65	11.25	0.00	0.00	-90.29	1.00
RIM	5	184.89	11.49	0.00	0.00	-87.29	1.00
PBM_RIM	6	185.51	12.11	0.00	0.00	-86.55	1.00
pc1	2	173.95	0.00	1.00	0.40	-84.95	0.40
pc1_pbm	3	174.60	0.64	0.73	0.29	-84.24	0.68
MI	2	175.52	1.56	0.46	0.18	-85.73	0.87
EMI	2	177.53	3.57	0.17	0.07	-86.74	0.93
hclust	3	178.12	4.17	0.12	0.05	-86.00	0.98
dep	2	182.00	8.04	0.02	0.01	-88.97	0.99
ETB	2	182.08	8.13	0.02	0.01	-89.01	1.00
PBM	2	184.65	10.69	0.00	0.00	-90.29	1.00
RIM	5	185.13	11.17	0.00	0.00	-87.41	1.00
PBM_RIM	6	185.79	11.83	0.00	0.00	-86.68	1.00

Table S8: Pooled estimates for each model in our exploratory analysis after multiple imputation. Estimates are beta coefficients in logistic regression models. Within- and between- imputation variance, total variance, and standard error (SE) are reported here.

outcome	model	predictor	est	within	between	total	SE
trust	pc1	pc1	-1.22	0.04	0	0.04	0.19
trust	pc1__pbm	pc1	-1.24	0.04	0	0.04	0.20
trust	pc1__pbm	condition	0.39	0.12	0	0.12	0.34
trust	mi	MI	0.89	0.03	0	0.03	0.17
trust	dep	depend	-0.59	0.04	0	0.04	0.20
trust	pbm	condition	0.11	0.09	0	0.09	0.30
trust	rim	insecure	0.41	0.03	0	0.03	0.16
trust	rim	need	0.44	0.04	0	0.05	0.21
trust	rim	depend	-0.51	0.04	0	0.05	0.21
trust	rim	wealth	-0.33	0.04	0	0.04	0.19
trust	pbm__rim	condition	0.25	0.11	0	0.11	0.33
trust	pbm__rim	insecure	0.41	0.03	0	0.03	0.16
trust	pbm__rim	need	0.41	0.04	0	0.05	0.21
trust	pbm__rim	depend	-0.54	0.05	0	0.05	0.22
trust	pbm__rim	wealth	-0.32	0.04	0	0.04	0.19
trust	EMI__ETB	EMI	1.05	0.04	0	0.04	0.20
trust	EMI__ETB	ETB	-0.13	0.08	0	0.08	0.28
trust	EMI	EMI	1.07	0.04	0	0.04	0.19
trust	ETB	ETB	-0.47	0.08	0	0.08	0.28
check	pc1	pc1	0.68	0.04	0	0.04	0.20
check	pc1__pbm	pc1	0.70	0.04	0	0.04	0.20
check	pc1__pbm	condition	-0.47	0.16	0	0.16	0.40
check	mi	MI	-0.60	0.04	0	0.04	0.20
check	dep	depend	0.41	0.06	0	0.06	0.25
check	pbm	condition	-0.32	0.15	0	0.15	0.38
check	rim	insecure	-0.23	0.04	0	0.04	0.19
check	rim	need	0.06	0.04	0	0.04	0.20
check	rim	depend	0.38	0.07	0	0.07	0.26
check	rim	wealth	0.25	0.05	0	0.05	0.23
check	pbm__rim	condition	-0.49	0.16	0	0.16	0.40
check	pbm__rim	insecure	-0.23	0.04	0	0.04	0.19
check	pbm__rim	need	0.09	0.05	0	0.05	0.21
check	pbm__rim	depend	0.45	0.07	0	0.07	0.27
check	pbm__rim	wealth	0.23	0.06	0	0.06	0.24
check	EMI__ETB	EMI	-0.45	0.03	0	0.04	0.19
check	EMI__ETB	ETB	0.38	0.15	0	0.15	0.39
check	EMI	EMI	-0.50	0.03	0	0.03	0.18
check	ETB	ETB	0.57	0.17	0	0.17	0.41

Table S9: Imputed dataset 1. Logistic regression models for trust outcomes based on exploratory models (MI, EMI, ETB, EMI+ETB, and dependence on livestock only), and on confirmatory models (after imputation; condition, and scaled measures of household food insecurity, need, wealth, and dependence on livestock).

	Dependent variable:									
	trust									
	(1)	(2)	(3)	(4)	(5)	(6)	(7)	(8)	(9)	(10)
pc1	−1.22***	−1.23***								
	(0.19)	(0.19)								
conditionprestige		0.39						0.11		0.24
		(0.34)						(0.30)		(0.33)
MI			0.85***							
			(0.17)							
EMI				1.06***	1.04***					
				(0.19)	(0.20)					
ETB					−0.13				−0.47	
					(0.28)				(0.29)	
insecure							0.42**			0.42**
							(0.16)			(0.16)
need							0.44*			0.41
							(0.21)			(0.21)
depend						−0.56**	−0.47*			−0.51*
						(0.20)	(0.20)			(0.21)
wealth							−0.31			−0.30
							(0.19)			(0.19)
Note:	*p<0.05; **p<0.01; ***p<0.001									

Table S10: Imputed dataset 1. Logistic regression models for fact-checking outcomes based on exploratory models (MI, EMI, ETB, EMI+ETB, and dependence on livestock only), and on confirmatory models (after imputation; condition, and scaled measures of household food insecurity, need, wealth, and dependence on livestock).

	Dependent variable:									
	check									
	(1)	(2)	(3)	(4)	(5)	(6)	(7)	(8)	(9)	(10)
pc1	0.68***	0.70***								
	(0.20)	(0.20)								
conditionprestige		−0.48						−0.32		−0.49
		(0.40)						(0.38)		(0.40)
MI			−0.61**							
			(0.20)							
EMI				−0.51**	−0.46*					
				(0.18)	(0.19)					
ETB					0.37				0.57	
					(0.40)				(0.42)	
insecure							−0.23			−0.23
							(0.19)			(0.19)
need							0.06			0.09
							(0.20)			(0.22)
depend						0.39	0.37			0.44
						(0.24)	(0.25)			(0.27)
wealth							0.25			0.24
							(0.23)			(0.24)
Note:								*p<0.05; **p<0.01; ***p<0.001		

Table S11: Imputed dataset 2. Logistic regression models for trust outcomes based on exploratory models (MI, EMI, ETB, EMI+ETB, and dependence on livestock only), and on confirmatory models (after imputation; condition, and scaled measures of household food insecurity, need, wealth, and dependence on livestock).

	Dependent variable:									
	trust									
	(1)	(2)	(3)	(4)	(5)	(6)	(7)	(8)	(9)	(10)
pc1	-1.22^{***}	-1.24^{***}								
	(0.19)	(0.19)								
conditionprestige		0.39						0.11		0.24
		(0.34)						(0.30)		(0.33)
MI			0.93^{***}							
			(0.17)							
EMI				1.09^{***}	1.07^{***}					
				(0.19)	(0.20)					
ETB					-0.11				-0.45	
					(0.28)				(0.28)	
insecure							0.41^{**}			0.41^{**}
							(0.16)			(0.16)
need							0.44^{*}			0.41
							(0.21)			(0.21)
depend						-0.58^{**}	-0.49^{*}			-0.53^{*}
						(0.20)	(0.21)			(0.22)
wealth							-0.35			-0.35
							(0.19)			(0.19)
Note:	$^{*}p<0.05$; $^{**}p<0.01$; $^{***}p<0.001$									

Table S12: Imputed dataset 2. Logistic regression models for fact-checking outcomes based on exploratory models (MI, EMI, ETB, EMI+ETB, and dependence on livestock only), and on confirmatory models (after imputation; condition, and scaled measures of household food insecurity, need, wealth, and dependence on livestock).

	Dependent variable:									
	check									
	(1)	(2)	(3)	(4)	(5)	(6)	(7)	(8)	(9)	(10)
pc1	0.67***	0.69***								
	(0.20)	(0.20)								
conditionprestige		−0.48						−0.32		−0.48
		(0.40)						(0.38)		(0.40)
MI			−0.62**							
			(0.20)							
EMI				−0.50**	−0.45*					
				(0.18)	(0.19)					
ETB					0.37				0.55	
					(0.39)				(0.41)	
insecure							−0.23			−0.23
							(0.19)			(0.19)
need							0.07			0.10
							(0.20)			(0.21)
depend						0.40	0.38			0.45
						(0.24)	(0.26)			(0.27)
wealth							0.24			0.23
							(0.23)			(0.23)
Note:							*p<0.05; **p<0.01; ***p<0.001			

Table S13: Imputed dataset 3. Logistic regression models for trust outcomes based on exploratory models (MI, EMI, ETB, EMI+ETB, and dependence on livestock only), and on confirmatory models (after imputation; condition, and scaled measures of household food insecurity, need, wealth, and dependence on livestock).

	Dependent variable:									
	trust									
	(1)	(2)	(3)	(4)	(5)	(6)	(7)	(8)	(9)	(10)
pc1	−1.23***	−1.24***								
	(0.19)	(0.20)								
conditionprestige		0.38						0.11		0.24
		(0.34)						(0.30)		(0.33)
MI			0.88***							
			(0.17)							
EMI				1.06***	1.04***					
				(0.19)	(0.20)					
ETB					−0.12				−0.47	
					(0.28)				(0.28)	
insecure							0.41**			0.41**
							(0.16)			(0.16)
need							0.47*			0.44*
							(0.21)			(0.21)
depend						−0.59**	−0.49*			−0.53*
						(0.20)	(0.21)			(0.22)
wealth							−0.36			−0.35
							(0.19)			(0.19)
Note:							*p<0.05; **p<0.01; ***p<0.001			

Table S14: Imputed dataset 3. Logistic regression models for fact-checking outcomes based on exploratory models (MI, EMI, ETB, EMI+ETB, and dependence on livestock only), and on confirmatory models (after imputation; condition, and scaled measures of household food insecurity, need, wealth, and dependence on livestock).

	Dependent variable:									
	check									
	(1)	(2)	(3)	(4)	(5)	(6)	(7)	(8)	(9)	(10)
pc1	0.69***	0.71***								
	(0.20)	(0.20)								
conditionprestige		−0.47						−0.32		−0.48
		(0.40)						(0.38)		(0.40)
MI			−0.60**							
			(0.20)							
EMI				−0.49**	−0.45*					
				(0.18)	(0.19)					
ETB					0.36				0.55	
					(0.38)				(0.40)	
insecure							−0.23			−0.23
							(0.19)			(0.19)
need							0.04			0.07
							(0.19)			(0.20)
depend						0.42	0.39			0.46
						(0.25)	(0.26)			(0.27)
wealth							0.24			0.23
							(0.23)			(0.23)
Note:										*p<0.05; **p<0.01; ***p<0.001

Table S15: Imputed dataset 4. Logistic regression models for trust outcomes based on exploratory models (MI, EMI, ETB, EMI+ETB, and dependence on livestock only), and on confirmatory models (after imputation; condition, and scaled measures of household food insecurity, need, wealth, and dependence on livestock).

	Dependent variable:									
	trust									
	(1)	(2)	(3)	(4)	(5)	(6)	(7)	(8)	(9)	(10)
pc1	-1.23^{***}	-1.24^{***}								
	(0.19)	(0.20)								
conditionprestige		0.40						0.11		0.27
		(0.34)						(0.30)		(0.33)
MI			0.89^{***}							
			(0.17)							
EMI				1.08^{***}	1.06^{***}					
				(0.19)	(0.20)					
ETB					-0.14				-0.49	
					(0.28)				(0.28)	
insecure							0.40^{*}			0.40^{*}
							(0.16)			(0.16)
need							0.40			0.37
							(0.20)			(0.20)
depend						-0.63^{**}	-0.55^{**}			-0.59^{**}
						(0.20)	(0.21)			(0.22)
wealth							-0.29			-0.29
							(0.19)			(0.19)
Note:								$^{*}p<0.05$; $^{**}p<0.01$; $^{***}p<0.001$		

Table S16: Imputed dataset 4. Logistic regression models for fact-checking outcomes based on exploratory models (MI, EMI, ETB, EMI+ETB, and dependence on livestock only), and on confirmatory models (after imputation; condition, and scaled measures of household food insecurity, need, wealth, and dependence on livestock).

	Dependent variable:									
	check									
	(1)	(2)	(3)	(4)	(5)	(6)	(7)	(8)	(9)	(10)
pc1	0.68***	0.70***								
	(0.20)	(0.20)								
conditionprestige		−0.47						−0.32		−0.49
		(0.40)						(0.38)		(0.40)
MI			−0.59**							
			(0.20)							
EMI				−0.48**	−0.43*					
				(0.18)	(0.19)					
ETB					0.40				0.58	
					(0.40)				(0.41)	
insecure							−0.23			−0.22
							(0.19)			(0.19)
need							0.09			0.12
							(0.21)			(0.22)
depend						0.41	0.39			0.46
						(0.25)	(0.26)			(0.27)
wealth							0.27			0.25
							(0.23)			(0.24)
Note:	*p<0.05; **p<0.01; ***p<0.001									

Table S17: Imputed dataset 5. Logistic regression models for trust outcomes based on exploratory models (MI, EMI, ETB, EMI+ETB, and dependence on livestock only), and on confirmatory models (after imputation; condition, and scaled measures of household food insecurity, need, wealth, and dependence on livestock).

	Dependent variable:									
	trust									
	(1)	(2)	(3)	(4)	(5)	(6)	(7)	(8)	(9)	(10)
pc1	-1.23^{***}	-1.25^{***}								
	(0.20)	(0.20)								
conditionprestige		0.40						0.11		0.25
		(0.34)						(0.30)		(0.33)
MI			0.91^{***}							
			(0.17)							
EMI				1.09^{***}	1.06^{***}					
				(0.19)	(0.20)					
ETB					-0.14				-0.49	
					(0.28)				(0.28)	
insecure							0.41^{**}			0.42^{**}
							(0.16)			(0.16)
need							0.46^{*}			0.44^{*}
							(0.21)			(0.21)
depend						-0.60^{**}	-0.52^{*}			-0.55^{*}
						(0.20)	(0.21)			(0.22)
wealth							-0.35			-0.34
							(0.19)			(0.19)

Note: *p<0.05; **p<0.01; ***p<0.001

Table S18: Imputed dataset 5. Logistic regression models for fact-checking outcomes based on exploratory models (MI, EMI, ETB, EMI+ETB, and dependence on livestock only), and on confirmatory models (after imputation; condition, and scaled measures of household food insecurity, need, wealth, and dependence on livestock).

	Dependent variable:									
	check									
	(1)	(2)	(3)	(4)	(5)	(6)	(7)	(8)	(9)	(10)
pc1	0.66**	0.68***								
	(0.20)	(0.20)								
conditionprestige		−0.47						−0.32		−0.48
		(0.40)						(0.38)		(0.40)
MI			−0.61**							
			(0.20)							
EMI				−0.51**	−0.45*					
				(0.18)	(0.19)					
ETB					0.39				0.58	
					(0.39)				(0.41)	
insecure							−0.23			−0.23
							(0.19)			(0.19)
need							0.04			0.07
							(0.19)			(0.21)
depend						0.41	0.38			0.45
						(0.25)	(0.26)			(0.27)
wealth							0.25			0.23
							(0.23)			(0.23)
Note:								*p<0.05; **p<0.01; ***p<0.001		

7 Material and ideational culture clusters

In our results, both in the exploratory analyses and in the AICc tables shown above, we separated variables belonging to ideational (TB, or *traditional beliefs*) and material categories (MI, EMI, denoting *market integration* and *empirical market integration*,[6] respectively). Variables in each category were all included in our PCA, and therefore comprise subsets of the PCA variables (i.e., loading on PC1, the *acculturation* variable; figure 2 in the main text).

It is worth exploring here, in more detail, interrelationships among these covariates of PC1 (acculturation). Specifically, missionization and education are often thought to be largely responsible for the fact that Maasai values and norms are largely shifting away from traditional beliefs (TB). There was a roughly equal split among Christians (51%) vs. traditional Maasai believers (49%) across regions, but it is difficult to keep Christianity *completely* separate from the changing material conditions (MI); missionization, along with non-government organizations funded from Western sources (often Christian), has emphasized an increasing focus on educational development, infrastructure among the villages such as Eluwai, and contributed an influx of cash and resources in the area.

7.1 Variation in trust outcomes for each cluster as predictor

Comparing the models in our exploratory analysis to each other, and to the confirmatory models, we showed that market integration and empirical market integration each predicted higher trust (market integration: $\beta = 0.87$, $SE = 0.34$; empirical market integration: $\beta = 1.1$, $SE = 0.39$) and lower fact-checking (market integration: $\beta =$ -0.59, $SE = 0.39$; empirical market integration: $\beta =$ -0.5, $SE = 0.37$), whereas traditional beliefs weakly predicted lower trust ($\beta =$ -0.46, $SE = 0.56$) and higher fact-checking ($\beta = 0.56$, $SE = 0.83$). These effects were larger those in the RIM, but neither were as large as the effect of acculturation (trust: $\beta = 1.2$, $SE = 0.4$; check: $\beta =$ -0.67, $SE = 0.42$).

7.2 Correlations among clusters and other predictors

Here, we show how market integration, empirical market integration, traditional beliefs, and other aspects of acculturation (PC1), along with outcome variables, are correlated with each other. In general, (an *a priori* measure of) market integration was higher in the southern region (mean = 0.84) than in the northern region (mean = -0.51; $t = 12.8$, $p = 6 \times 10^{-27}$). Market integration and empirical market integration each strongly correlated with acculturation (market integration: $r = 0.72$, empirical market integration: $r =$ -0.82), and traditional beliefs moderately correlated with acculturation ($r = 0.25$). Market integration, empirical market integration, and traditional beliefs were similarly intercorrelated. Although Christianity weakly correlated with market integration ($r = 0.17$) and empirical market integration ($r = 0.23$), it was not correlated with traditional beliefs ($r =$ -0.04). This seems to suggest that acculturation is largely driven by market integration, but less driven by traditional beliefs, and, more interestingly, Christianity is largely *independent* of changes in market integration, traditional beliefs, and acculturation.

It is worth noting, however, that our traditional beliefs cluster was partially driven by variation in herd sizes, a clearly material domain. As shown in the main text, these were collapsed into a single cluster strictly as a result of our hierarchical clustering analysis. This leads to the compelling question of why was this material domain so tightly linked to variation in our ideational variables. The answer could be relevant either to traditional beliefs and values, or to locational differences relative to the market and towns near the southern region, specifically as a consequence of more private land and less available grazing land.

[6] To re-emphasize here, as we discuss in the main text results, *empirical market integration* refers to the market integration variable that resulted from our hierarchical clustering analysis. We distinguish this from the *market integration* variable, which, as discussed in our main methods section, was constructed prior to our exploratory analyses based on proxy measures of cash wealth/reliance and market purchases for subsistence.

7.2.1 Correlation matrix

We reported that market integration and empirical market integration were strongly associated with acculturation, traditional beliefs was moderately associated with acculturation, and that market integration, empirical market integration, and traditional beliefs were similarly intercorrelated with each other. We also noted that Christianity weakly correlated with market integration and empirical market integration, but it was not correlated with traditional beliefs. See figure S12 for a correlation matrix showing these associations. Note that although traditional beliefs and Christianity were not correlated with each other, each of these variables weakly to moderately correlated with other variables listed here, including acculturation. It is also worth pointing out that out of their covariates, the strongest associations for each Christianity and traditional beliefs were seen with acculturation.

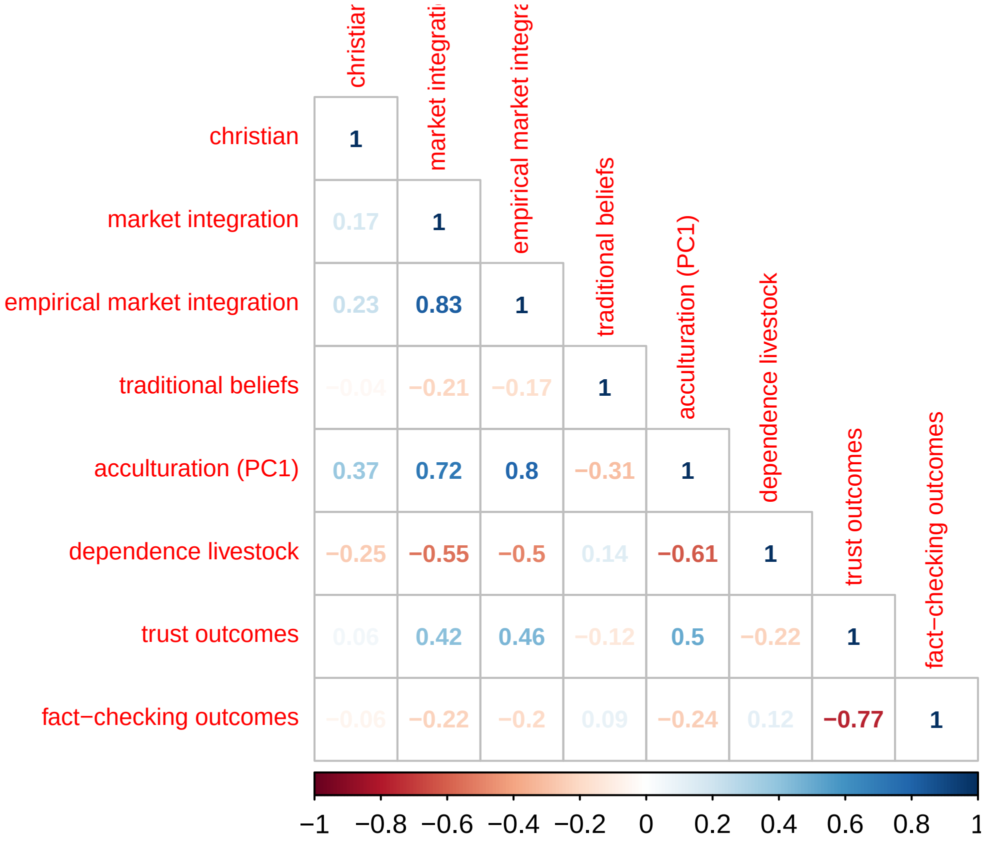

Figure S12: Correlation matrix for Christianity, market integration (both a priori, MI and empirically driven MI, EMI), traditional beliefs (ETB), PC1, dependence on livestock, and trust and fact-checking outcomes.

8 Sex differences in the PCA

We found little-to-no meaningful sex differences in the PCA results. Specifically, PC1 values were not systematically different among males and females, but it is worth noting that the variance and skew on PC2 were higher for males than they were for females (figure S13). This is unsurprising, as we interpreted PC2 as largely corresponding to certain aspects of wealth (e.g., number of wives) and household size (see figure 2 in main text), which in Maasai culture, vary among males much more than they do among females (see also Spencer 1965).

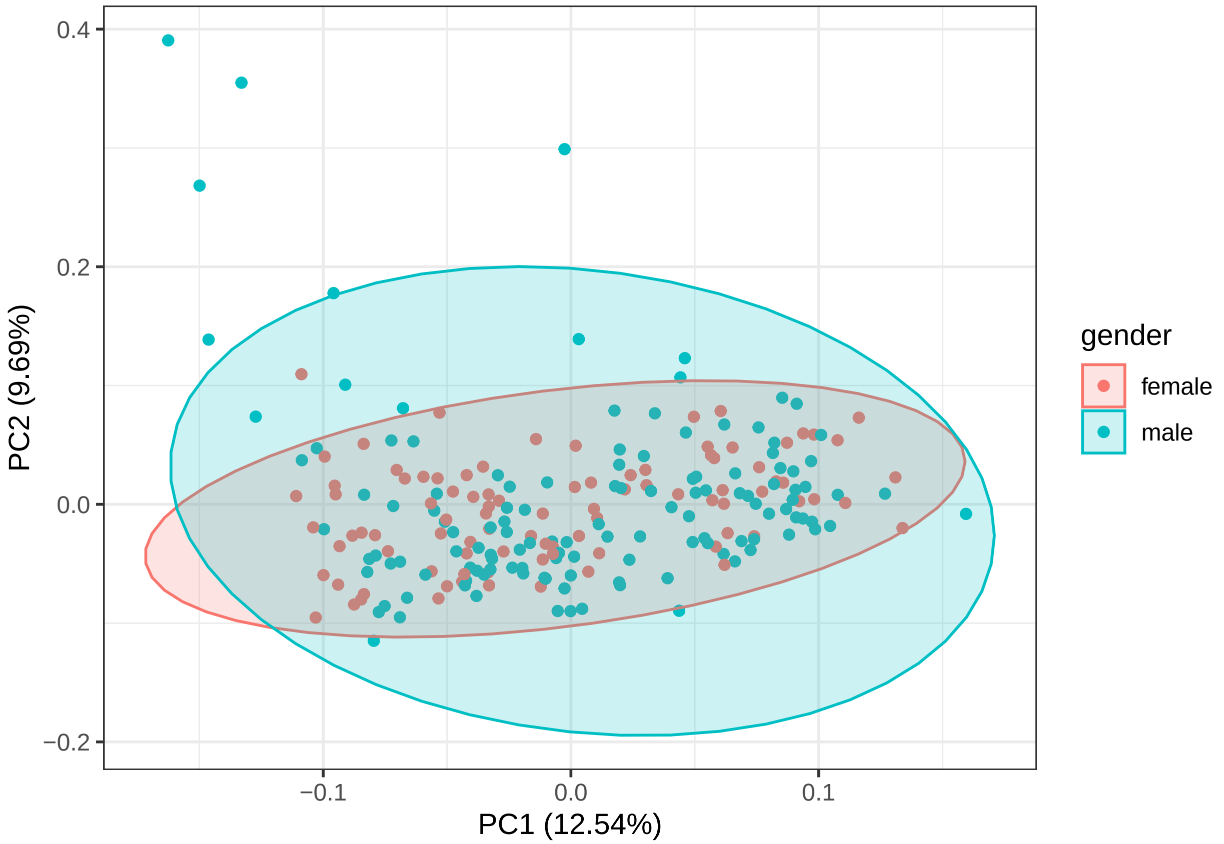

Figure S13: Biplot of PCA results from the exploratory analysis, with participant sex indicated by color. Similar to our main results, we interpreted PC1 as relating to acculturation and PC2 as relating to household size.
